# Spatial chromosome organization and adaptation of the radiation-resistant extremophile *Deinococcus radiodurans*

**DOI:** 10.1016/j.jbc.2024.108068

**Published:** 2024-12-10

**Authors:** Qin-Tian Qiu, Cai-Yun Zhang, Zhi-Peng Gao, Bin-Guang Ma

**Affiliations:** Hubei Key Laboratory of Agricultural Bioinformatics, College of Informatics, Huazhong Agricultural University, Wuhan, China

**Keywords:** chromatin remodeling, transcription regulation, genome structure, bacterial genetics, energy metabolism, chromosome organization, ultraviolet irradiation, nucleoid-associated protein, *Deinococcus radiodurans*

## Abstract

Radiation-resistant *Deinococcus radiodurans* is an extremophilic microorganism capable of withstanding high levels of ionizing radiation and chemical mutagens. It possesses remarkable DNA repair capability and serves as a model organism for studying stress resistance mechanisms. However, our understanding of the spatial chromosome organization of this species remains limited. In this study, we employed chromosome conformation capture (3C) technology to determine the 3D genome structure of *D. radiodurans* and to further investigate the changes of chromosome conformation induced by ultraviolet (UV) irradiation. We observed that UV irradiation reduced short-range chromosome interactions, and smaller chromosomal interaction domains (CIDs) merged to form larger CIDs. Integrating transcriptomic data analysis, we found that the majority of upregulated differentially expressed genes were significantly enriched near specific CID boundaries. Specifically, we comprehensively elucidated that the nucleoid-associated protein DrEbfC as a global regulatory factor for gene expression, may modulate the efficiency of relevant metabolic pathways by altering the local chromosome structure, thereby influencing the physiological state of the bacterium. Overall, our study revealed the chromosome conformations of *D. radiodurans* under different conditions and offered valuable insights into the molecular response mechanism of this extremophile to survival stresses.

For both eukaryotes and prokaryotes, the carrier of genetic information, DNA, combines with proteins to form chromosome which requires a high degree of compression to fit into a cellular space thousands of times smaller than the length of the stretched DNA molecule. The highly compacted chromosomes form organized structures to meet the functional requirements of gene expression, DNA replication, and chromosome segregation (1). Over the past decade, significant advancements in our understanding of bacterial genome dynamics and interactions have been achieved through the application of chromosome conformation capture (3C)-based techniques (2, 3, 4, 5, 6, 7, 8, 9). Presently, chromosome contact maps of each studied bacterial genome have shown clear chromosomal interaction domains (CIDs), similar to the topologically associating domains found in eukaryotes (10, 11). Chromosome regions within the same CID tend to interact more frequently than inter-domain regions, and the directionality index (DI) or insulation score can be used to identify such domains based on this characteristic. Except for the smaller-sized domains (15–30 kb) found in *Mycoplasma pneumoniae*, CIDs in other bacteria typically range from 30 to 400 kb (5). The specific mechanisms underlying CID formation are still unclear. It is suggested that the formation of CID may be related to the transcription process, DNA supercoiling, and macromolecular crowding. It has been observed that CID boundaries almost disappear after treatment with the transcription inhibitor Rifampicin in *Bacillus subtilis*, suggesting that long and active transcriptional regions contribute to CID boundary formation (2, 12). In addition, nucleoid-associated proteins (NAPs) play diverse roles in the process of bacterial nucleoid organization (13, 14). In *Escherichia coli*, various NAPs act on chromosomal structures at local or global scales. For example, HU and Fis proteins facilitate long-range DNA interactions, while the MatP protein restricts chromosomal interactions within a 280 kb region at the chromosome replication terminus region (6). A recent study found that Rok protein in *B*. *subtilis* forms large (Mb range) and well-defined anchored chromosomal loops, physically isolating large regions of chromosome (15).

*Deinococcus radiodurans* exhibits an exceptional capacity to withstand extreme environmental conditions, characterized by a radiation tolerance approximately 30-fold greater than that of *E*. *coli* and several thousand-fold greater than that of human cells (16). Generally, radiation can disrupt the structure and function of chromosomal DNA through physical effects and secondary oxidative damage, resulting in a substantial accumulation of single- or double-stranded DNA breaks, which culminate in cellular demise. *D. radiodurans* possesses 4 to 10 genome copies within a single cell, and studies have suggested that its robust environmental adaptation capabilities are largely attributed to its multiple genome copies or redundant genetic information (17). Despite extensive research on DNA damage repair mechanism under environmental stress (18, 19, 20, 21, 22), the investigation of chromosomal 3D structures in response to stress in *D. radiodurans* is still lacking. Furthermore, compared to radiation-sensitive bacteria, *D. radiodurans* possesses highly compacted nucleoid structures that remain unchanged even after exposure to high doses of radiation (23). NAPs may play a role in this high degree of nucleoid compaction. The types and quantities of NAPs in *D. radiodurans* are species-specific (24). Based on amino acid sequence analysis, a few known NAPs have homologs in *D. radiodurans*, including HU (*dr_a0065*), DnaA (*dr_0002*), Dps (with *dr_2263* referred to as Dps1 and *dr_b0092* as Dps2), Lrp (*dr_1894*, *dr_0200*), and EbfC (*dr_0199*) (25, 26, 27, 28).

EbfC (*erp*-binding factor, chromosomal), also known as YbaB, is a less-studied NAP but widely distributed in prokaryotes (29). It was first discovered in *Borrelia burgdorferi* and characterized as a site-specific DNA-binding protein (30). Homologs of EbfC found in *E. coli* and *Hemophilus influenzae* also function as a transcriptional regulator and play roles in DNA repair. The EbfC protein in *D. radiodurans*, identified in 2012, is a novel member of NAPs. Preliminary experiments have shown that the DrEbfC protein localized in the nucleoid region of cells and constrained DNA supercoiling *in vitro* (27). So far, research on the YbaB/EbfC proteins has mainly focused on exploring their functions using molecular biological methods. From the perspective of 3D genome, further investigations are required to unravel the role of EbfC in the mechanism of chromosome organization.

In this study, the chromosome conformation captures with the deep sequencing (3C-seq) technique was applied to explore the structural features of *D. radiodurans* chromosomes, and the differences in chromosome 3D structure and gene expression before and after ultraviolet (UV) irradiation were analyzed combined with transcriptome data. Furthermore, the impact of DrEbfC protein inactivation on chromosomal contacts was elucidated, manifested by the reduction in local short-range interactions and changes in metabolic flux resulting from chromosome structural alterations. Findings in this research for the first time reveal the effects of an external stress (UV irradiation) and an internal cellular stress (the NAP DrEbfC deletion) on chromosome organization at the 3D genomics level and provide a new perspective for understanding the role of chromosome organization in the radiation resistance mechanism of *D. radiodurans*.

## Results

### Global conformation of the *D. radiodurans* genome

Chromosomal 3D structures reflect the regulatory functions of genome organization in cellular activities. In this work, we applied 3C-seq to the cells of *D. radiodurans* R1 strain in the mid-exponential phase to explore the 3D organization of its multipartite genome. Fig. S1 presents the coverage of sequencing reads along the genome position for 3C-seq samples. Replicate experiments conducted under various conditions present reproducible results, which are displayed by generating normalized contact map ratios (Fig. S2*A*) and correlation heatmap (Fig. S2*B*).

**Figure 1:**
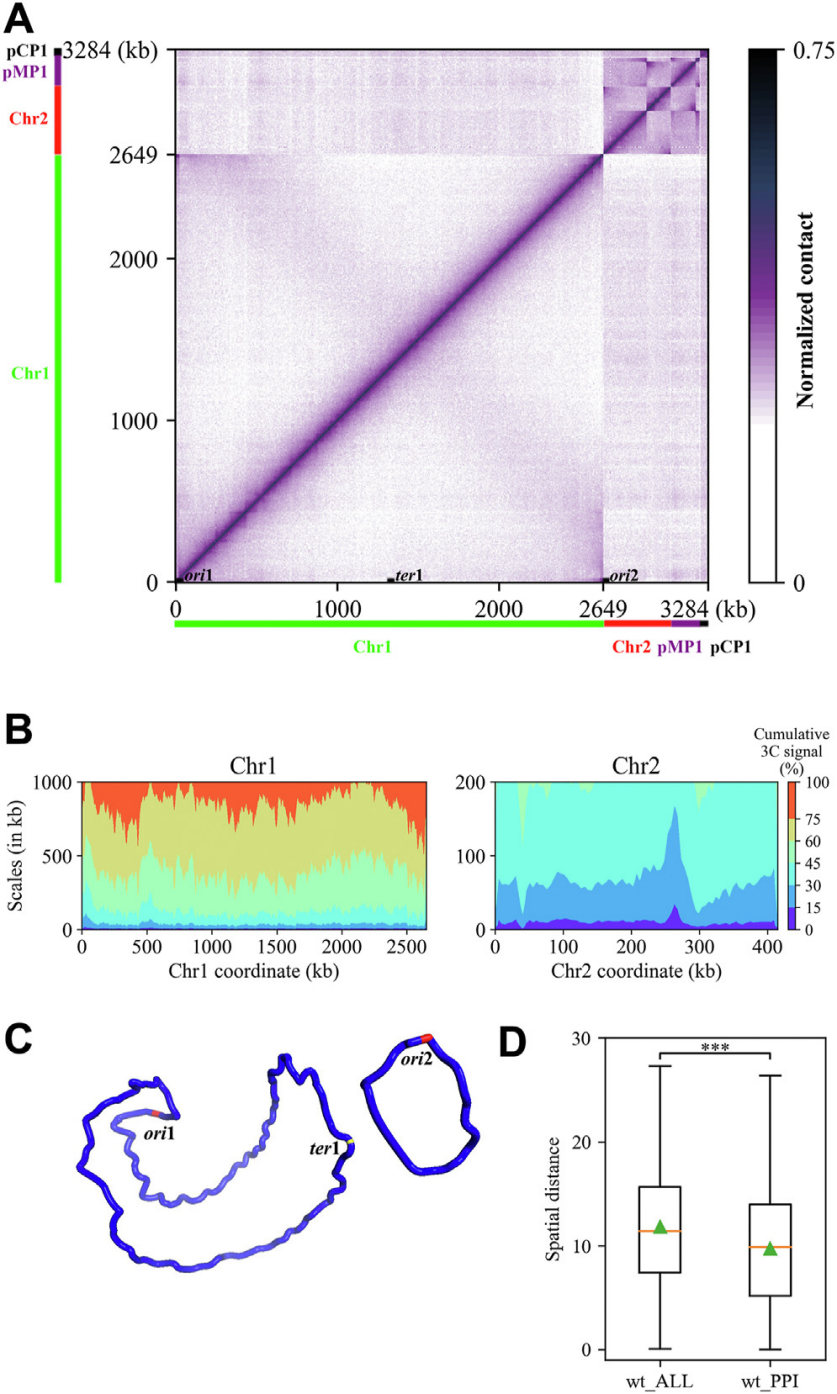
**Spatial organization of *D. radiodurans* replicons.***A*, normalized contact map displays contact frequencies of 5 kb bins across the genome of *D. radiodurans* R1 wild-type (WT) cells growing exponentially. The *x* and *y* axes represent genomic coordinates of each replicon. Chr1, Chr2, pMP1, and pCP1 are indicated by *green*, *red*, *purple*, and *black* bars, respectively. The positions of the origins (ori1 and ori2) and terminus (ter1) are indicated on the *x* axis. *B*, scalogram representation of the two chromosomes of *D. radiodurans*. Scalograms reflect relative compactness of the chromosome regions. The colored areas above each bin represent the fraction of the total cumulated contacts made by the bin with its flanking regions of increasing sizes (*dark blue*, 0–15%; *light blue*, 15–30%, …; *red*, 75–100%). Compact regions display small *blue* and large *red* areas. Loose regions display large *blue* and small *red* areas. *C*, the 3D model of the two chromosomes of *D. radiodurans*. The origins (*ori*1 and *ori*2) are marked as *red* and the terminus (*ter*1) is marked as *yellow*. *D*, boxplot of the spatial distance distribution between bin pairs in the 3D model. wt_ALL: all bin pairs in the genome; wt_PPI: the bin pairs containing protein-protein interaction (namely, one bin in a bin pair contains one of the two interacting proteins, and the other bin in the bin pair contains the other interacting protein). The orange line indicates the median of the box. The green triangle indicates the average value of the box. ∗∗∗: *p*-value < 0.001.

The genome of *D*. *radiodurans* consists of four replicons: the large chromosome Chr1 [2649 kb], the small chromosome Chr2 [412 kb], the large plasmid pMP1 [177 kb], and the small plasmid pCP1 [46 kb] (31). Figure 1*A* displays the normalized 5-kb contact map of chromosomes of wild-type cells, and it shows a strong diagonal signal, indicating a propensity for neighboring loci to interact with each other along the genome. Additionally, the contact map of Chr1 exhibits a faint secondary diagonal, reflecting weak inter-arm interactions between loci on the left and right arms of Chr1, and there seems no significant inter-arm interactions for Chr2 and the two plasmids (Fig. S3). Compared with other bacteria, it was found that the interaction pattern of Chr1 resembles that of *B*. *subtilis*, *Caulobacter crescentus*, and *Vibrio cholerae* Chr2 (32). To further interpret the contact map, scalograms were generated by calculating the sum of interaction frequencies within a certain range upstream and downstream of each bin on the chromosome, which provides insights into local chromosomal behaviors. This visualization tool has been employed to investigate the impact of NAPs on the local spatial compaction of the genome in *E. coli* (6). Figure 1*B* illustrates the uneven distribution of local compaction of *D*. *radiodurans* Chr1 and Chr2 at different scales.

Given the small size (in bp) of the two plasmids in *D*. *radiodurans*, we focused on modeling the 3D structures of Chr1 and Chr2 at a 5 kb resolution using the EVRC software (33). This software considers both intra-chromosomal and inter-chromosomal interactions, allowing us to reconstruct this multipartite genome. Figure 1*C* shows the obvious difference in the average structure of the two chromosomes. Specifically, Chr1 exhibits a boat-like shape with its replication origin (*ori*1) located at the bow of the boat in the 3D model, corresponding to the first bin (1183–1903 bp) on the genome. The arms of Chr1 align along the longitudinal axis and have a closer proximity. In contrast, Chr2 has a more circular shape, with a larger distance between the arms, and its replication origin (*ori*2) is also located in the first bin of the Chr2 genome. Previous work in *E. coli* has found that protein-protein interaction (PPI) correlates with DNA interaction on a genome-scale (34). To investigate this feature, we collected the PPI pairs (totally 2997) of *D*. *radiodurans* from the STRING database (35). Using the spatial distance from the chromosome 3D model, we found that DNA fragments involved in functional PPIs are spatially closer to each other (*p* = 3.6 × 10^−66^) (Fig. 1*D*). Importantly, this phenomenon was also observed when the environmental condition or physiological state of this organism changed (Fig. S4), highlighting the conservativeness of this chromosomal 3D feature.

### Analysis of chromosomal interaction domains (CIDs) in the 3D genome of *D. radiodurans*

**Figure 2:**
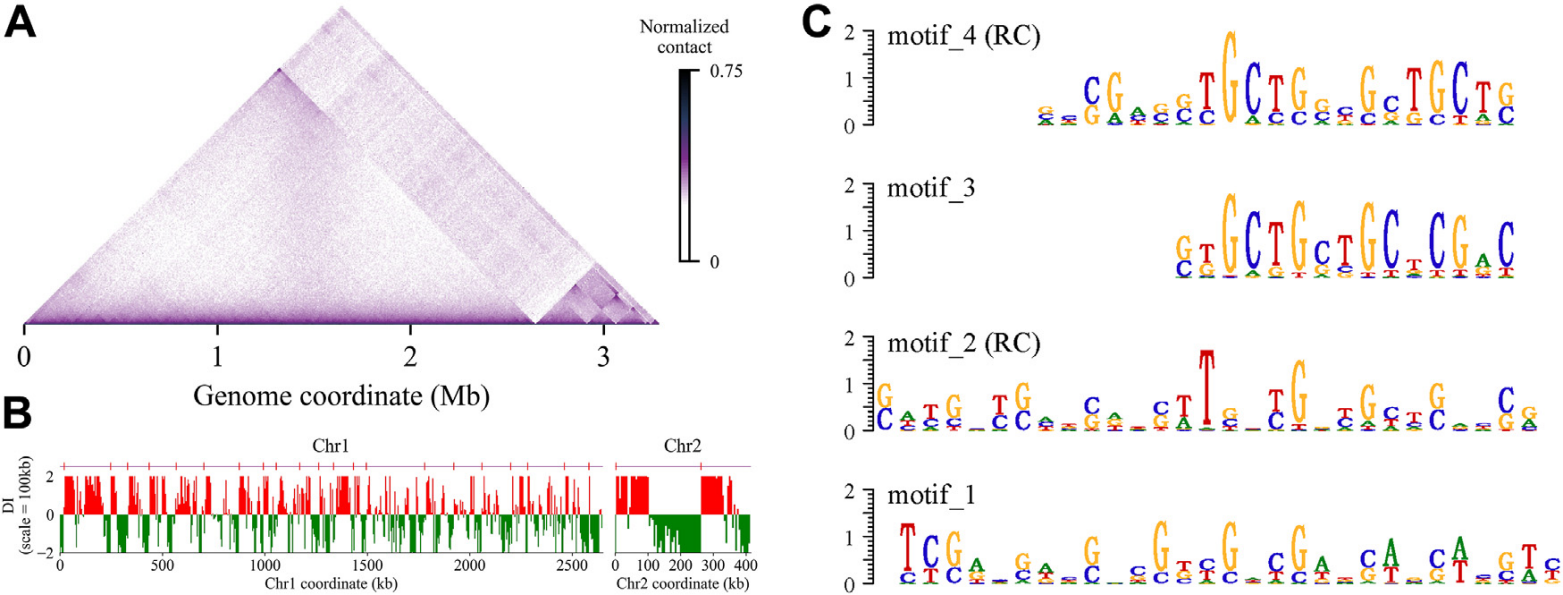
**CID analysis of *D. radiodurans* chromosomes at 5 kb resolution.***A*, normalized contact map for the genome rotated 45° clockwise. *B*, domain boundaries characterized for wild-type condition using DI analysis performed at a scale of 100 kb. Downstream (*red*) and upstream (*green*) biases are indicated. Significant DI boundaries defining CIDs are annotated with *red* vertical lines above the panel. *C*, motifs at CID boundaries discovered through MEME software. RC represents the motifs discovered on the reverse complementary chain.

The 3C contact map exhibits highly self-interacting regions or CIDs that appear as squares along the main diagonal (Fig. 1*A*). When the map is rotated 45° clockwise, it appears triangular (Fig. 2*A*). Using the directionality index (DI) at a scale of 100 kb, we identified a total of 23 CIDs in the wild-type cells (Chr1: 21, Chr2: 2) (Fig. 2*B*), ranging in size from 30 kb to 250 kb (average size: 101 kb), which is consistent with the CID scales observed in other bacteria (5). Further analysis of the guanine and cytosine (G  + C) content distribution within the internal and boundary regions of the CIDs in *D*. *radiodurans* was performed. The results reveal that CID boundaries have lower G  + C content, and 78.3% of the CID boundaries (18 out of 23) are lower than the average level of chromosomal G  + C contents. Moreover, the G  + C content of CID boundaries is significantly lower than the interior (Fig. S5), which has also been reported in other species (36, 37), suggesting a potential role of AT-rich DNA sequences in CID boundary formation. In view of the biased base composition of the CID boundary, we use the MEME software to search for conserved sequences (motifs) at these boundaries. Then the TOMTOM software was used to annotate and compare the motif sequences obtained, and it was found that some known transcription factor binding sites are significantly present in the boundary regions. For these motifs, visualization is present in Figure 2*C*, and detailed information is provided in Table S2. Some of them are also known binding motifs for NAPs involved in transcriptional regulation, implying the potential influence of NAPs on the establishment or maintenance of local chromosome structure.

Furthermore, we explored the function of genes located at CID boundaries. Although some genes are unannotated, KEGG pathway annotation of the remaining 468 genes reveals that 80.3% of them are involved in metabolic processes, followed by genetic information processing (9.4%) and environmental information processing (7.5%), with only 2.8% of the genes associated with cellular processes (Fig. S6*A*). Further GO enrichment analysis of CID boundary genes shows that the boundary genes are mainly enriched in the biological processes of metabolism and proton transmembrane transport. The gene products are predominantly located in the cytoplasm and ATPase complex, with molecular functions related to ATP binding, transport, and catalysis (Fig. S6*B*). These results reflect the important roles of CID boundary genes in the growth and metabolic processes of *D*. *radiodurans*.

### Interplay between transcription and local chromosome structure

**Figure 3:**
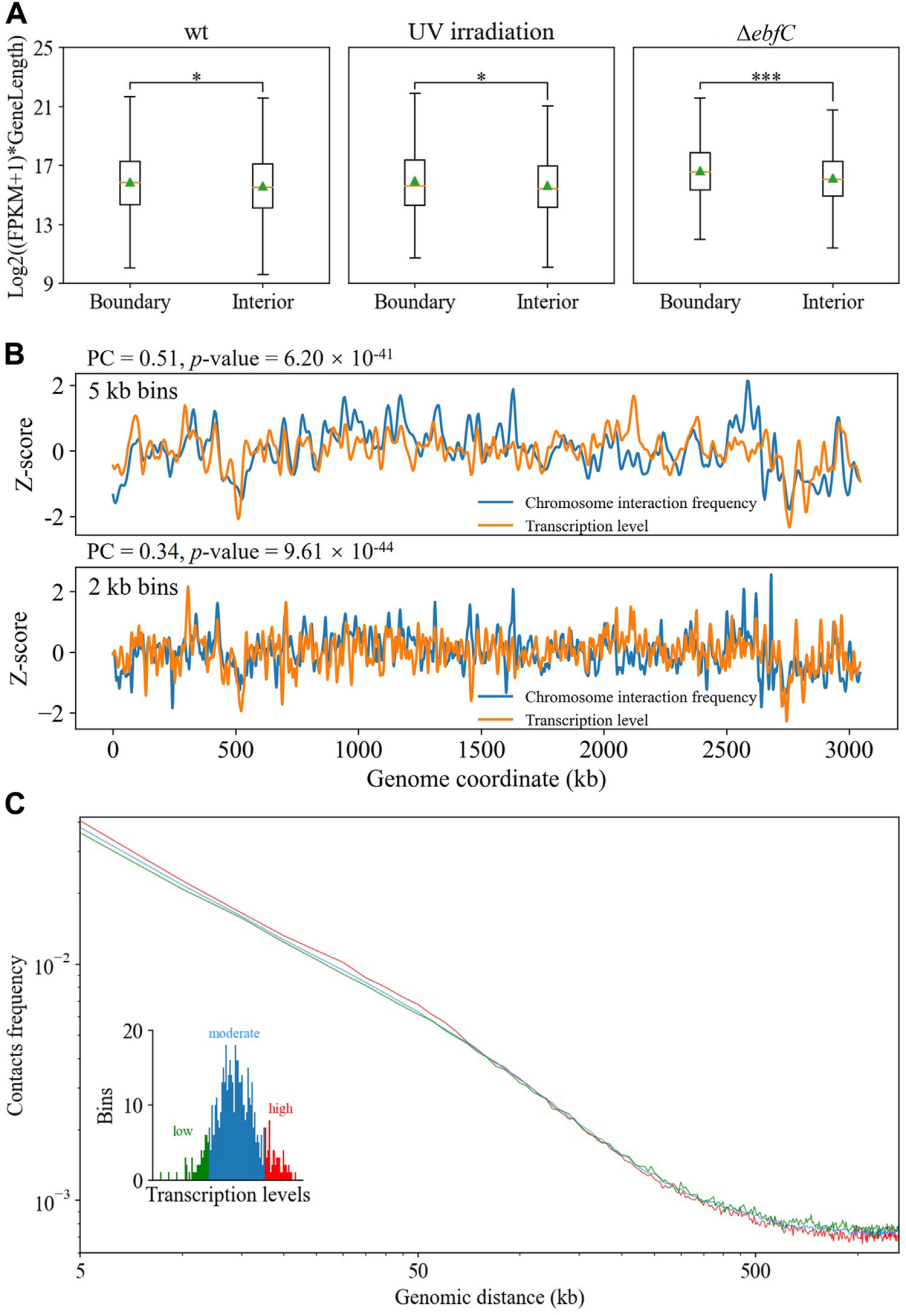
**Correlation between transcription and local chromosome structure.***A*, box plots of CID boundary and interior gene expression distributions corresponding to different samples. The *orange* line indicates the median of the box. The *green* triangle indicates the average value of the box. ∗∗∗, *p*-value < 0.001; ∗, *p*-value < 0.05. *B*, correlation between transcription level and short-range chromosome interaction frequency (diagonal value of interaction matrix) at 5-kb and 2-kb resolution. The abscissa denotes the location on the genome, while the ordinate represents the strength of the normalized signal value. The Pearson correlation (PC) between these two variables and the corresponding *p*-value is shown at the *top* of the graph. *C*, contact frequency as a function of genomic distance for genes categorized based on their expression levels: poorly expressed (*green*), moderately expressed (*blue*), or highly expressed (*red*). The *top* 10% and *bottom* 10% of gene expression levels were classified as highly expressed and poorly expressed, respectively, with the remaining categorized as moderately expressed. Inset: distribution of bin numbers for different transcription levels (at 5 kb resolution).

Previous studies have demonstrated that long and highly expressed transcripts in *B. subtilis* can drive local chromosome decompaction and promote spatial separation of gene flanking sequences, thereby reducing contact between DNA in neighboring domains to form CID boundaries (12). This phenomenon is also found in *D. radiodurans*. After obtaining gene expression profiles through transcriptome sequencing, we examined the lengths and transcriptional levels of genes at the CID boundary and interior. Figure 3*A* shows that long genes with active transcription significantly exist at CID boundaries, which holds true for both UV irradiation conditions and Δ*ebfC* mutation.

Moreover, we investigated the changes in DNA interaction frequency and transcriptional level along the genome. Figure 3*B* shows a modest but significant positive correlation between the two sets of signals at two binning sizes. Notably, similar correlations have been observed in contact maps of other bacteria species generated by different laboratories using different enzymes and cross-linking conditions (6), indicating that such a correlation is a conservative feature. Additionally, the relationship between interaction frequency and genome distance was plotted according to gene expression level (Fig. 3*C*), which shows that the bins containing highly expressed genes have higher interaction frequencies with nearby bins, and the interaction frequency decays fast with the increase of linear distance. This suggests that the transcription process may limit the mobility of gene loci, resulting in reduced long-range interactions and increased short-range interactions. However, compared to *E. coli* and *B*. *subtilis* (6), the effect in Figure 3*C* seems modest. Research has shown that unlike *E. coli*, where the genomic DNA is evenly distributed throughout the entire cell, the nucleoid of *D. radiodurans* is highly compacted and occupies relatively less space in the cell (38). We speculate that the densely packed nucleoid of *D. radiodurans* makes the separation of genomic regions with different gene expression levels less obvious.

### UV irradiation alters chromosomal interaction regions

**Figure 4:**
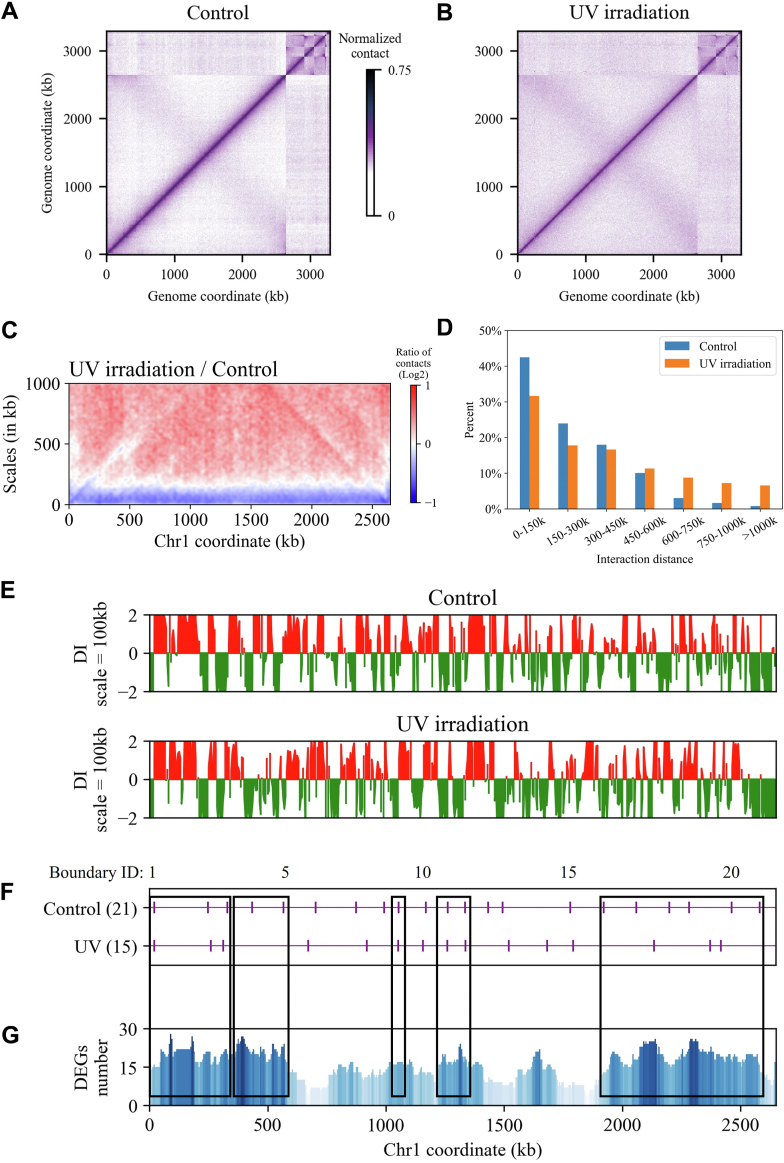
**Effect of UV irradiation on chromosome contacts and CID boundaries.***A* and *B*, normalized contact maps for the control group and UV irradiation condition, respectively. *C*, ratio (UV/Control) plot of the contact signals for each bin along Chr1. The *x*-axis indicates the position of the bin along the genome. The *y*-axis indicates the distance from the bin. A decrease or increase in contacts at the UV condition compared with the control is represented with a *blue* or *red* color, respectively. The *white* color indicates no difference between the two conditions. *D*, proportion of significant *cis*-interactions of Chr1. The significant *cis*-interactions are classified according to different distance ranges. The interaction distance is calculated based on the genomic coordinate in the circular genome. If the distance between the significant interacting loci exceeds half of the length of Chr1, the shorter arc length is considered as the interaction distance. The *x*-axis is the interval of different interaction distances; the *y*-axis is the proportion of significant interactions within the distance interval to the total significant interactions. *E*, DI analysis (100 kb) of Chr1 at Control and UV conditions, respectively. *F*, CID boundaries along the Chr1 are marked by *purple* vertical lines. *G*, distribution of upregulated DEGs in Chr1. The *black* boxes across the subplots *F* and *G* represent the CID boundaries with enriched upregulated DEGs.

To investigate the impact of UV irradiation on the chromosomal structure of *D. radiodurans*, we performed 3C experiments on cells exposed to 750 J/m^2^ UV irradiation for 5 min. Viability assay reveals a survival rate of approximately 76.6% under this UV dosage (39), indicating sufficient live cells for 3C experiments. Irradiation stress often triggers responses in DNA recombination repair and base excision repair pathways. Analysis of the normalized interaction matrix shows that UV irradiation led to weaker interaction (lighter color) along the main diagonal and stronger interaction (darker color) at other positions compared to the control group cells (Fig. 4, *A* and *B*). To further visualize the interaction difference, a ratio plot is used to show the interaction between each bin and its adjacent bins. The results indicate a reduction in short-range interactions and an increase in long-range interactions after UV irradiation (Fig. 4*C*). To confirm this phenomenon, we employed the Fit-Hi-C software to count significant interactions between pairs of bins at a 5 kb resolution across the whole genome. The results show that the interaction frequency of chromosome decreases with the increase of linear distance, with fewer significant short-range interactions (<450 kb) and more significant long-range interactions (>450 kb) after irradiation compared with control group cells (Fig. 4*D*). Furthermore, we identified 18 CIDs from the contact map of irradiated cells, ranging in size from 10 kb to 335 kb, with an average size of 139 kb. Five CIDs are conserved, and the larger CIDs observed in irradiated cells seem to be formed by the fusion of several smaller CIDs present in the control group cells (Fig. S7).

Meanwhile, we performed transcriptome analysis on the irradiated cells and identified 750 differentially expressed genes (DEGs) with a fold change threshold of 2. Among these, upregulated genes (544) account for 72.5% and are significantly enriched in DNA recombination repair, SOS response, and translation processes (39). To explore the relationship between DEGs, particularly upregulated genes and chromosomal structural changes, we analyzed the distribution of differentially upregulated genes on *D. radiodurans* Chr1 using a sliding step of 5 kb and a window size of 100 kb. The results, as shown in Figure 4, *E*–*G*, reveal that the distribution of differentially upregulated genes along the genome is not random but significantly enriched near certain CID boundaries. These special CID boundaries are mainly divided into two categories: the first consists of CID boundaries that are present in the control group cells but disappear after irradiation (*e.g.*, ID_4, 5, 16, 17, 18, 19, 20, 21); the second comprises the conserved CID boundaries (*e.g.*, ID_1, 2, 3, 9, 11, 12) in both irradiated and control group cells. Furthermore, GO enrichment results of CID boundary genes specific to the control group and the irradiation group were compared and analyzed (Fig. S8), and it was found that the function of boundary genes changed significantly. After UV irradiation, the specific CID boundary genes are mainly involved in the translation and ribosome assembly processes, and the protein products constitute the respiratory chain complex, ribosome subunit structure, and other components, and perform the functions of ribosome structure maintenance and rRNA binding. The damage repair pathways of *D. radiodurans* in response to UV irradiation require the substantial synthesis of relevant proteins and the generation of ATPs through respiration. These results collectively suggest that *D. radiodurans* employs a coordinated response of chromosomal organization and transcriptional regulation to cope with UV irradiation stress and minimize damage to the bacterial cell.

### DrEbfC is a global transcriptional regulator in *D. radiodurans*

**Figure 5:**
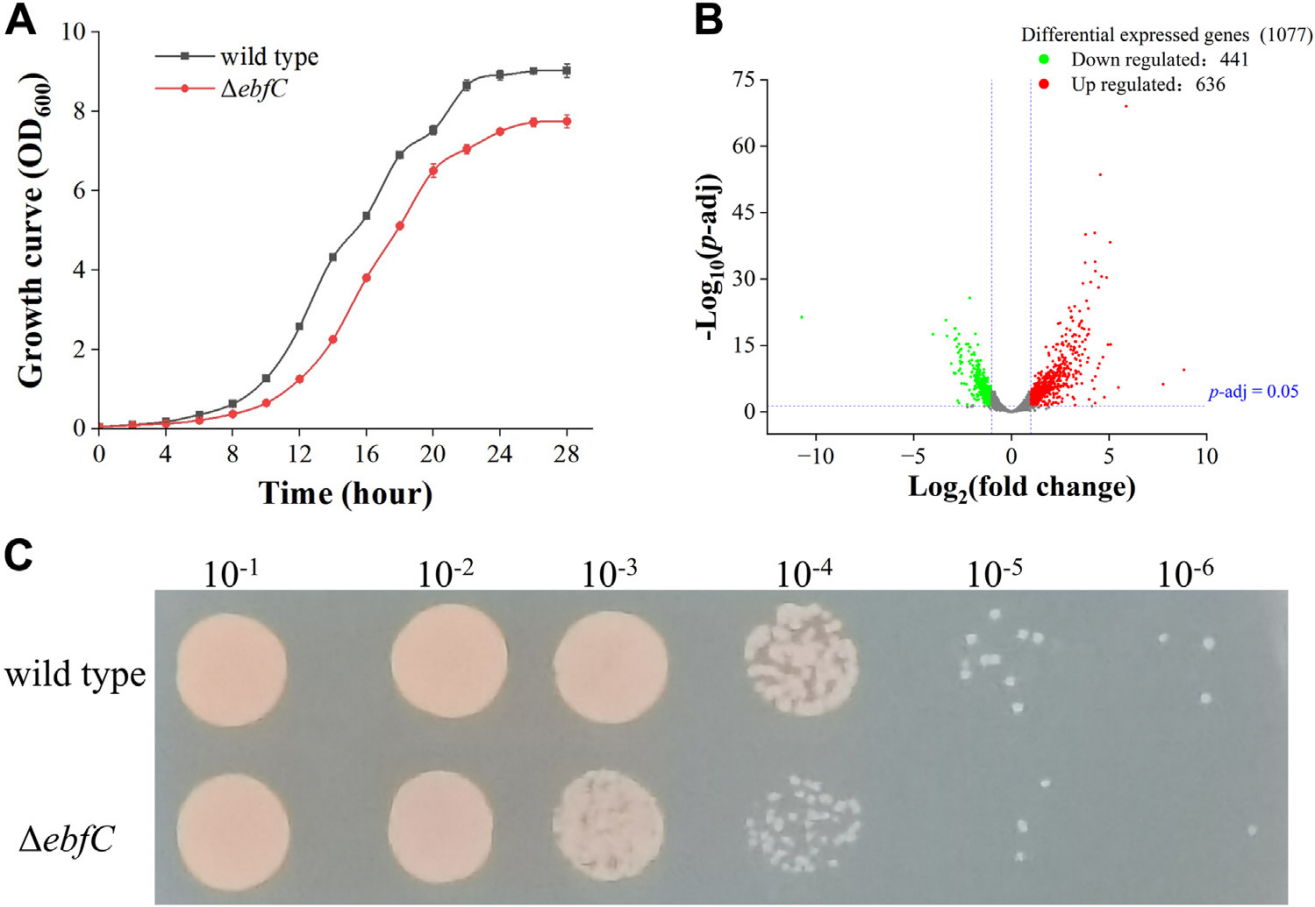
**Comparative analysis of phenotypes and transcriptional profiles between wild-type and Δ*ebfC* mutant strains.***A*, growth rate of wild-type and Δ*ebfC* strain in TGY. *B*, volcano plot of DEGs in the Δ*ebfC* strain compared with *D. radiodurans* wild type. Genes with an adjusted *p* < 0.05 are assigned as DEGs. *C*, susceptibility experiment of wild-type and Δ*ebfC* strains of *D. radiodurans* to H_2_O_2_. After incubating in a 50 mM hydrogen peroxide solution for 30 min, the diluted bacterial suspension was spotted onto TGY agar plates. The values above the picture represent the dilution ratio of the bacterial suspension.

The DrEbfC protein in *D. radiodurans*, encoded by the *dr_0199* gene, was initially characterized as a NAP with DNA-protective and histone-like properties, safeguarding the DNA from damage (27). Its homologs are almost universal in all bacteria, showing a high degree of sequence conservation (Fig. S9). Previous work through sequence analysis has shown that the DrEbfC protein homologous dimer adopts a “tweezer-like” structure (Fig. S10), where α-helices form the potential domain responsible for DNA binding (29). To study the role of this protein in *D. radiodurans*, we constructed the *dr_0199* deletion mutant in *D. radiodurans*. The mutant strain is viable, but compared with wild-type cells, the growth rate is moderately inhibited and the cell density during the stationary phase is lower than that of the wild-type (Fig. 5*A*).

Through RNA-seq analysis of the mutant strain, we identified a total of 1077 DEGs, with 636 upregulated genes and 441 downregulated genes compared to the wild-type (Fig. 5*B*). GO and KEGG pathway analyses indicate that the upregulated genes in the mutant are mainly associated with environmental stress response, DNA recombination, regulation of metabolic activities, catalytic activities, and DNA binding processes (Fig. S11, *A* and *B*). Notably, several DNA damage repair-related genes (*e.g.*, *ddrA*, *ddrB*, *ddrC*, *ddrD*, *pprA*) regulated by PprI/DdrO proteins show significantly increased expression levels in the mutant (Table S3). This observation suggests that the absence of the DrEbfC protein might lead to cellular stress, implying a potential role of this NAP in DNA protection. The PprI/DdrO-mediated regulatory system has been recognized as a unique transcriptional inhibition and removal system of *D. radiodurans* in response to DNA damage. Under environmental stress conditions, PprI, a metal-dependent protease, is activated to specifically cleave the C-terminal region of the DdrO, abolishing its DNA binding activity, and resulting in the expression of DNA damage repair genes that are inhibited by the DdrO (40). Enrichment analysis of the downregulated genes in the mutant reveals their involvement in vital cellular processes closely related to respiration, translation, RNA metabolism, and electron transfer (Fig. S11, *C* and *D*), corroborating the observed slow growth phenotype of the mutant. Additionally, genes encoding oxidative stress resistance proteins, such as catalase (KatA, KatE, DR_RS14330) and superoxide dismutase (SodA, DR_RS07910), show significant down-regulation in the mutant (Table S4). Among the three known catalases in *D. radiodurans*, DR1998 is the most important one, exhibiting a high activity of 68,800 U/mg and serving as a more effective scavenger of hydrogen peroxide compared to the other two enzymes (41). The transcription levels of *pdxT* and *pdxS* genes, encoding pyridoxine biosynthesis proteins, are also significantly decreased by more than threefold in the mutant strain. The enzymes of pyridoxal 5′-phosphate biosynthesis participate in the biosynthesis of vitamin B6, acting as an efficient quencher of singlet oxygen and potential antioxidant (42). The downregulation of these genes indicates a reduced resistance of Δ*ebfC* mutant to oxidative damage, which is further confirmed by the hydrogen peroxide sensitivity assay of the mutant strain, as shown in Figure 5*C*.

### The role of DrEbfC protein in chromosome organization and adaptation

**Figure 6:**
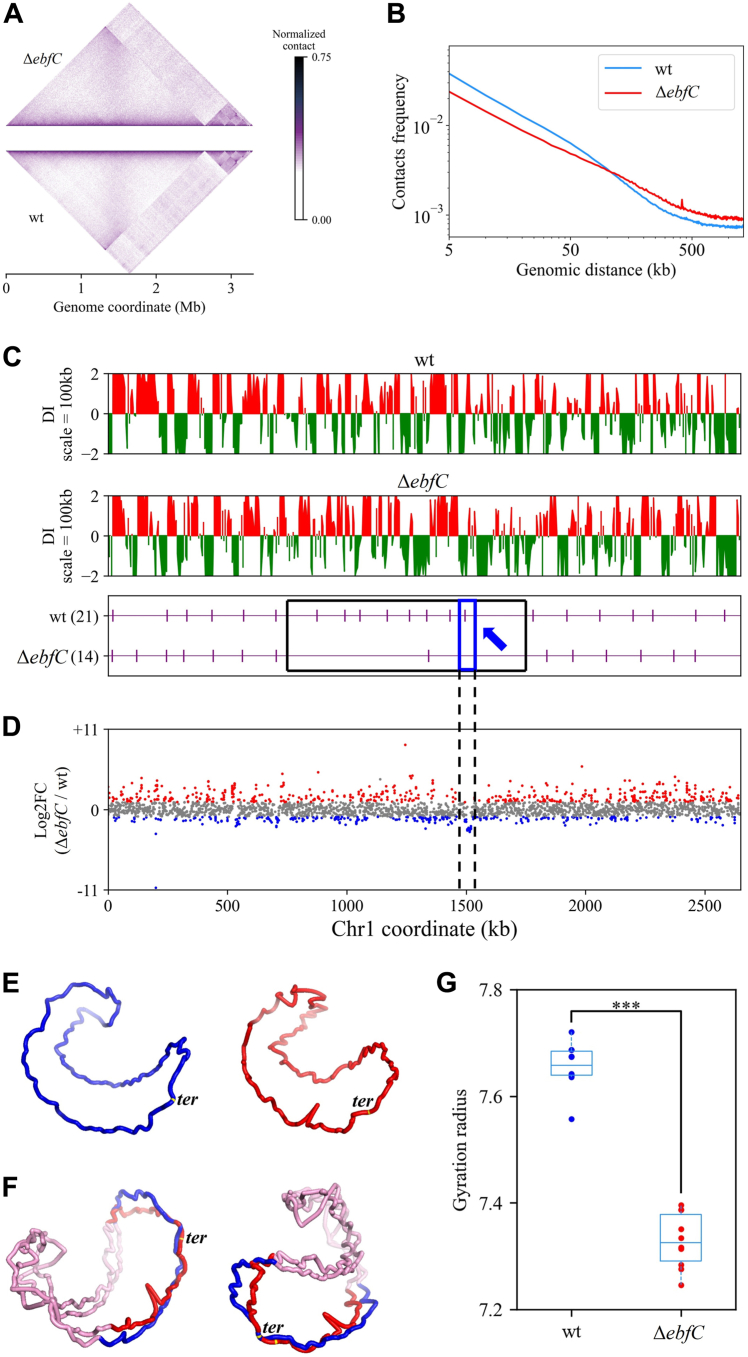
**The role of DrEbfC protein in *D. radiodurans* chromosome organization.***A*, symmetric halves of the normalized contact maps of Δ*ebfC* mutant (*top*) and wild-type (*bottom*) cells are aligned after clockwise rotation. *B*, plot of chromosome interaction frequency as a function of genomic distance. *C*, CID distribution on Chr1 at 5 kb resolution of wild-type and Δ*ebfC* strains, respectively. The *purple* vertical lines below represent CID boundaries along the Chr1. The CID boundaries lost in the Δ*ebfC* strain are highlighted by the *black* box. The area with boundary loss and gene expression changes is marked by the *blue arrow*. *D*, the distribution of DEGs of wild-type and Δ*ebfC* strains on Chr1. *Red* and *blue dots* represent upregulated and downregulated DEGs (fold change > 2 and FDR < 0.05), respectively. *Grey* dots represent genes with no significant change in expression. *E*, the models of Chr1 for wild-type (*blue*) and Δ*ebfC* mutant (*red*). *F*, comparison of the chromosome 3D structure near the *ter* region with missing CID boundaries (*blue* for wild-type and *red* for Δ*ebfC* mutant, shown in two different perspectives). Other regions of the chromosome are colored in *pink*. *G*, boxplot for the gyration radius distribution of the Chr1 3D structure modes of wild-type and Δ*ebfC* strains. A smaller gyration radius indicates a more compact structure. To reduce the impact of randomness in structure modeling, 10 models are generated for each strain for calculation. The difference is significant (∗∗∗, *p*-value < 0.001).

The 3C-seq analysis of wild-type and Δ*ebfC* cells shows that the interaction matrices seem similar overall (Fig. 6*A*). However, the plot of interaction frequency as a function of distance exhibits a significant reduction in short-range interactions in the mutant compared to the wild-type, extending up to approximately 100 kb (Fig. 6*B*). Moreover, we used DI to calculate the degree of interaction preference of each bin on Chr1. Although the overall correlation of interaction preference between wild-type and mutant is relatively high (*r* = 0.63, *p* = 1.6 × 10^−60^), indicating some CID boundaries remain stable, a complete loss of seven CID boundaries around the region of 750 to 1750 kb (near the *ter* region) in the Δ*ebfC* mutant is observed (Fig. 6*C*). More visually, the reconstructed structure of Chr1 shows that the Δ*ebfC* mutant has more overall bending than the wild-type (Fig. 6*E*). There is also a significant difference in chromosome structure near the *ter* region where the CID boundaries are lost in the mutant (Fig. 6*F*). To quantitatively characterize the changes in spatial structure, we calculated the gyration radius for the reconstructed structure models of the wild-type and mutant Chr1. The results show that the gyration radius is significantly smaller in the mutant than in the wild-type (Fig. 6*G*). Additionally, we evaluated two quantitative metrics based on 3D structural models: global compactness (GC) and local compactness (LC) (43). Higher values in these metrics indicate a more compact or condensed structure. Fig. S12, *A* and *B* show that both GC and LC values (for scales > 50 kb) are higher in the structural model of Chr1 in the mutant compared to the wild-type. These findings suggest a more compact 3D chromosome structure in the mutant strain. Our analyses indicate that DrEbfC protein promotes local short-range interactions in the genome, and may play an important role in maintaining interactions between loci in the *ter* region.

To explore whether the 3D structure changes affect gene expression, we analyzed the DEGs corresponding to the *ter* region. We found a significant difference in transcription levels between the mutant and wild-type on Chr1, where about 30% of the genes show significant changes (Fig. 6*D*). Specifically, in the region of 1480 to 1523 kb, CID boundary exists only in the wild-type and disappears in the mutant (Fig. 6*C*). The expression of a group of genes corresponding to this region is significantly reduced (Table 1), and the products encoded are mainly subunits constituting NADH-quinone oxidoreductase which is involved in the electron transport chain and plays a crucial role in cellular respiration and energy production. These results suggest a potential causal relationship between chromosome structure change and gene expression alteration, implying coordinated adaptation of the mutant to survival stress.

**Table 1:** A group of genes in the bule arrow region of Chr1 in Figure 6*C*

Gene ID	Position (bp)		Product (NADH-quinone oxidoreductase)	Down-regulated expression (log2Foldchange)
DR_RS07640	1,506,995	1,508,473	subunit N	−2.72
DR_RS07645	1,508,474	1,509,898	subunit M	−2.89
DR_RS07650	1,509,948	1,511,879	subunit L	−2.80
DR_RS07655	1,511,886	1,512,188	subunit NuoK	−2.67
DR_RS07660	1,512,190	1,512,807	subunit J	−2.77
DR_RS07665	1,512,804	1,513,340	subunit NuoI	−2.60
DR_RS07670	1,513,512	1,514,705	subunit NuoH	−2.61
DR_RS07675	1,514,705	1,516,897	subunit NuoG	−2.52
DR_RS07680	1,517,019	1,518,353	subunit NuoF	−2.86
DR_RS07685	1,518,350	1,518,973	subunit NuoE	−3.08
DR_RS07690	1,518,940	1,519,416	hypothetical protein	−2.94
DR_RS07695	1,519,446	1,520,651	subunit D	−2.89
DR_RS07700	1,520,648	1,521,322	subunit C	−2.54
DR_RS07705	1,521,319	1,521,864	subunit B	−2.69
DR_RS07710	1,521,971	1,522,306	subunit A	−2.30

### Chromosome adaptation leads to slow growth *via* metabolic flux redistribution

**Figure 7:**
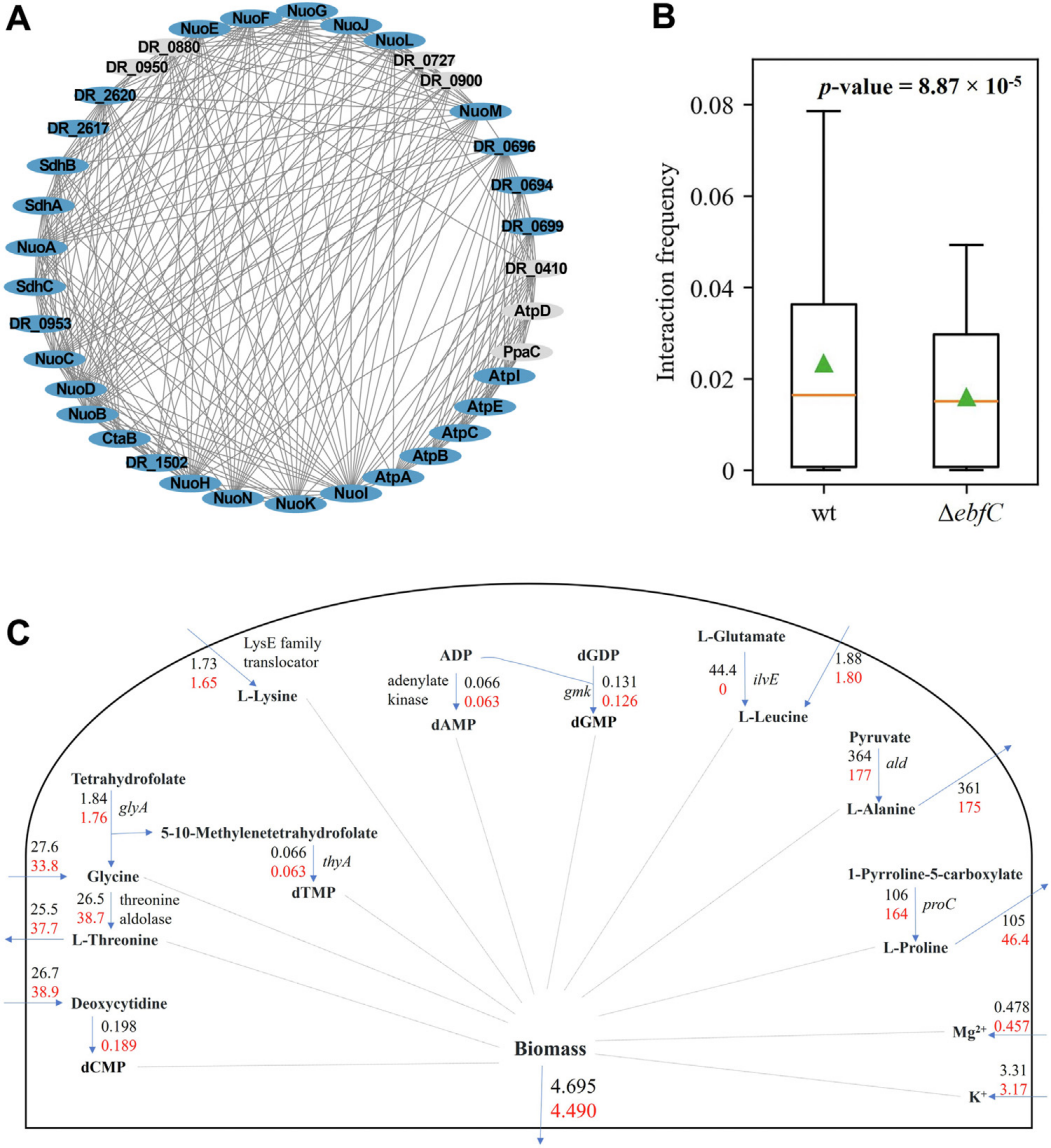
**Effect of DrEbfC protein on oxidative phosphorylation pathway and metabolic flux.***A*, the PPI network between proteins in the oxidative phosphorylation pathway of *D. radiodurans*, with *blue* and *gray* nodes representing genes with down-regulated expression and no significant change, respectively. *B*, comparison of the interaction frequency distribution between genes in the oxidative phosphorylation pathway between wild type and Δ*ebfC* strains. *C*, schematic diagram of biomass generation in the genome scale metabolic network of *D. radiodurans*. The direction of the *blue* arrow indicates the generation or transport of substances to the cytoplasm or extracellular space. The *black* and *red* values represent the reaction fluxes of wild-type and Δ*ebfC* mutant strains, respectively.

We explored the functions of these genes listed in Table 1 by querying the KEGG database, and found that this group of genes is involved in the oxidative phosphorylation pathway (dra00190: Oxidative phosphorylation). Using the STRING database, we searched for the PPI network related to this pathway in *D. radiodurans*, and visualized the network in Figure 7*A*. Upon further analysis of the nodes within this network, we observed that, except for the group of genes in Table 1, the expression levels of most other genes in this pathway were also significantly reduced in the Δ*ebfC* mutant. Moreover, given the characteristic of bacterial gene expression, where transcription and translation occur simultaneously due to a lack of cell nucleus (44, 45), the spatial positions of protein products are near to the positions of gene loci. Statistical analysis of the interaction matrix derived from 3C data reveals that the interaction frequencies between genes in this pathway decrease for the mutant (Fig. 7*A*), indicating an increase in spatial distance between these genes, *i.e.*, the spatial positions of the corresponding protein products become further apart in the mutant.

According to the theory of metabolic channeling, in enzymatic reactions occurring within organisms, the metabolites are transferred directly from the active site of one enzyme to the next, without diffusing into the surrounding solution. This reaction mechanism depends on the interaction between enzymes, such as the formation of multi-enzyme complexes or co-localization in space, which can significantly enhance reaction efficiency and metabolic flux (46, 47, 48). In the Δ*ebfC* mutant, the enzymes (proteins) involved in the oxidative phosphorylation pathway are downregulated in expression level (Table 1 and Fig. 7*A*), and meanwhile, the spatial distances between these proteins are increased due to the alteration of chromosome structure (Fig. 7*B*); these two factors may affect the metabolic fluxes in this pathway. Therefore, we further constructed a genome-scale metabolic model of *D. radiodurans* combined with transcriptome data constraints of the two strains, and conducted flux balance analysis. The results showed that the biomass optimization value of the Δ*ebfC* mutant was lower than that of the wild-type (Fig. 7*C*), which is consistent with the slower growth rate of the mutant. We speculate that DrEbfC protein may affect the efficiency of related metabolic reactions through dual changes in chromosome spatial structure and transcriptional regulation, thereby regulating the physiological state of the bacterium, manifested as slower growth (Fig. 5*A*).

## Discussion

Owing to the maturation of methodology, 3C/Hi-C techniques have been applied in more and more bacteria to investigate their genome organization and gene regulation over the past decade. In this study, we explored the 3D chromosomal architecture of the extremophile *D. radiodurans* and investigated the effects of UV irradiation on genome organization. Integrating transcriptome data and metabolic model, we also elucidated the role of DrEbfC as a NAP in gene expression and chromosomal organization.

We present genome-wide DNA interactions for all four replicons of *D. radiodurans* at a 5 kb resolution. Among them, contact maps of the two chromosomes (Chr1, Chr2) show similar interaction domains, but different inter-arm interactions patterns (Fig. S3). Chr1 displays secondary diagonal interactions, while Chr2 does not, indicating different folding states for these two chromosomes. On the other hand, a clear contact signal was observed between Chr2 and pMP1 (Fig. S13). Upon genomic sequence alignment, a highly similar nucleotide sequence (∼1340 nt) was identified between Chr2 and pMP1 at this location. Furthermore, according to the Z-curve profile predicted by Ori-Finder 2022 (49), the replication termination region of Chr2 is also in close proximity to this location. Studies on *E. coli* suggested that termination of replication is a highly intricate process, rigorously regulated to prevent potentially lethal DNA transactions (50). Collision of the replication fork in the terminus region may result in single-stranded and double-stranded DNA ends that instigate recombination (50). Therefore, we speculate that in the wild-type cells of *D. radiodurans*, the convergence of the replication fork resulting in heightened genomic instability may facilitate a recombination event between the highly similar segments on Chr2 and pMP1, showing a pronounced preferential contact between Chr2 and pMP1 in the contact map.

The mechanism of coordinated replication between chromosomes is of interest in other studied bacteria with multipartite genomes. In *V. cholerae*, the Ori region of Chr2 strongly interacts with the *crtS* region on Chr1, while the terminus regions of both Chr1 and Chr2 also exhibit preferential contacts (3). This is consistent with the mechanism by which two dissimilarly sized circular chromosomes of *V. cholerae* coordinate replication initiation but terminate replication at the same time. In *Agrobacterium tumefaciens*, the Ori regions of all four replicons are bundled together, resulting in an interaction pattern between the replicons that resembles a butterfly shape (9). To show the interactions between the two chromosomes of *D. radiodurans*, we positioned the Ori region at the center of each chromosome (Fig. S14). Unfortunately, we did not observe preferential contact between the origins or other regions on Chr1 and Chr2. It has been proposed that having multiple replicons enables faster genome replication and gives these bacteria the advantage of adapting quickly when switching hosts or environments (51). *D. radiodurans* also has multiple replicons and exhibits strong environmental adaptability, warranting further investigation into how these replicons coordinate and maintain genome stability within the bacterium.

The structural maintenance of chromosome (SMC) protein complex plays a pivotal role in shaping the global architecture of chromosomes, and extensive literature has elucidated the functions of numerous bacterial SMC proteins and their homologs (4, 6, 52). In this study, although the *smc* gene was not knocked out, transcriptome analysis of UV irradiation condition and Δ*ebfC* strain found that there were no significant differences in *smc* gene expression compared to the wild type. Notably, the absence of a homolog of the ScpA protein (53), one of the subunits that constitute the SMC complex in *D. radiodurans*, possibly contributes to the faint secondary diagonal of the contact map. Previous studies have shown that the SMC-ScpAB holo-complex can tether the two arms of the chromosome together and promote chromosome compaction and separation (52).

*D. radiodurans* has long been an ideal model organism for studying DNA damage repair processes following irradiation. We observed that the frequency of short-range interaction decreased after UV irradiation, but CIDs could still be detected using DI analysis. CIDs reduced and fused from smaller to larger domains by eliminating boundaries in the irradiated cells. This phenomenon reflects the adaptation of chromosome 3D structure of to environmental stress to reduce its damage to the organism. Combined with transcriptome analysis, we found that the number of upregulated genes was more than twice that of downregulated genes, with the upregulated genes predominantly located near specific CID boundaries in the genome. CID boundaries often correspond to long and active transcripts in *D. radiodurans*, which has also been demonstrated in *B. subtilis* (12). We conjecture that highly expressed long genes may more readily influence the preference of local interaction, affecting contacts between neighboring structures.

We investigated the role of DrEbfC as an NAP in *D. radiodurans* using a knockout inactivation strategy. Phenotypic experiments reveal that the Δ*ebfC* mutant is viable but exhibits slower growth and increased sensitivity to oxidative stress compared to the wild-type, which aligns well with previous findings conducted by Wang *et al.* (27). Transcriptomic analysis of the Δ*ebfC* mutant indicates down-regulation of genes involved in respiration and translation, providing molecular insights into the impact of DrEbfC as a regulatory factor on bacterial physiological activities. The DrEbfC protein has similar properties to other NAPs and can regulate gene expression by controlling chromosome topology (54, 55, 56). We identified a region of Chr1 in the Δ*ebfC* mutant where changes in chromosomal structure resulted in the down-regulation of a group of genes involved in the oxidative phosphorylation pathway. From the perspective of metabolic channeling, the increased spatial distances between enzymes involved in the successive reactions within this metabolic pathway, along with the reduced expression level of these enzymes in the Δ*ebfC* mutant, may lead to a redistribution of metabolic flux, thereby slowing down the energy production, ultimately resulting in a delayed growth phenotype of the mutant. While further experimental evidence is needed to support the metabolic regulatory mechanism of this protein, the exploratory results in this work have expanded our understanding of the chromosome behaviors of this extremophilic microorganism in response to survival stresses.

## Experimental procedures

### Strain, growth conditions and treatment

All strains and plasmids used in this study are listed in Table S1. *D. radiodurans* strains were grown at 30 °C in tryptone glucose yeast extract (TGY) liquid media or on agar plates (0.5% tryptone, 0.1% glucose, and 0.3% yeast extract). The cultures were grown to OD_600_ = 3.0 (middle exponential). For UV irradiation, the experiment was performed as reported previously (39).

### Construction of the *D. radiodurans* Δ*ebfC* strain

The Δ*ebfC* (*dr_0199*) strain was constructed by a tripartite ligation method, as described previously (57). Briefly, the DNA fragment upstream of *dr_0199* was amplified by PCR using the primers Δ*dr_0199*-p1 and Δ*dr_0199*-p2, which was digested with BamHI (Table S1). The DNA fragment downstream of *dr_0199* was amplified by PCR using the primers Δ*dr_0199*-p3 and Δ*dr_0199*-p4, which were digested with HindIII (Table S1). The digested fragments were ligated to a kanamycin resistance gene that was digested with BamHI and HindIII previously (Table S1). After the triplet ligation product was transformed into the *D. radiodurans* wild-type R1 strain, the mutant colonies were then selected on TGY plates containing 20 μg/ml kanamycin, and confirmed by genomic PCR using two pairs of primers (Δ*dr_0199*-p1 and Δ*dr_0199*-p4, Δ*dr_0199_test*-F and Δ*dr_0199_test*-R) and DNA sequencing.

### Growth curves and phenotypic assay

To measure the growth curve, the wild-type *D. radiodurans* R1 and Δ*ebfC* strains were pre-cultured to OD_600_ = 3.0 at 30 °C with shaking at 220 rpm/min, and then 1 ml of culture was transferred into 100 ml of fresh TGY medium without antibiotics. OD_600_ values were monitored at 2 h intervals until a point was reached where there was no longer a significant increase in the measured values. The final growth curve corresponding to the mean of three independent experiments was plotted using Origin 2023 software. To observe phenotypes under H_2_O_2_ treatment, the wild-type *D. radiodurans* R1 and Δ*ebfC* strains were pre-cultured to OD_600_ = 3.0, then treated with 50 mM H_2_O_2_ for 30 min. After the reaction, the mixture was diluted in a gradient and plated on a TGY plate. The experiment was repeated three times.

### Transcriptome data analysis

Bacterial cells were pre-cultured in TGY to an OD_600_ = 3.0, harvested by centrifugation at 120,00*g* for 3 min, and stored at −80 °C. Cryopreserved cells were dispatched to Wuhan Frasergen Bioinformatics Co., Ltd, where RNA extraction, cDNA library construction, and on-machine sequencing procedures were carried out. The sequencing standard was paired-end 150 bp, and the sequencing platform was MGISEQ-2000. After removing the adapter sequences and low-quality sequences from the raw reads, clean reads were obtained for mapping to the *D. radiodurans* R1 genome (GenBank assembly accession GCA_000008565.1) with Hisat2 Aligner (58). The mapped reads of each sample were applied to quantify the expression levels using StringTie (59) with a reference-based approach. Differential expression of genes was normalized and calculated by DEseq2 algorithm (60). Genes with expression level changes more than twofold increased or reduced with adjusted *p*-value < 0.05 were considered as differentially expressed genes (DEGs).

### 3C library construction and sequencing

3C experiments were carried out following the same procedure as described previously (6). Briefly, the genomic DNA from the bacteria was crosslinked with 7% formaldehyde for 1 h and quenched with a final concentration of 0.25 M glycine for 20 min at 4 °C. The fixed cells were lysed by incubation with lysozyme at 37 °C for 30 min. The reaction was quenched by adding 10% SDS and incubating for 5 min at 65 °C. 50 μl of lysed cells were then transferred in one EP tube containing 450 μl of digestion mix (355 μl of double distilled water, 50 μl of 10 × rCutSmart Buffer, 25 μl of 25% Triton X-100, and 20 μl of 10 U/μl HpaII enzyme). DNA was digested for 10 h at 37 °C with shaking at 200 rpm/min. For ligation, the reaction was mixed with 7945 μl of double distilled water, 1000 μl of 10 × T4 DNA Ligase Reaction Buffer (NEB), 50 μl of 20 mg/ml BSA (TaKaRa), 500 μl of 20% Triton X-100, and 5 μl of 400 U/μl T4 DNA ligase (NEB). The ligation reaction was incubated for 4 h at 16 °C with occasional inversion of the tube (every 1 h). After the ligation was completed, the reaction was mixed with 100 μl of 0.5 M EDTA and 50 μl of 20 mg/ml proteinase K (TransGen) and incubated at 58 °C for 4 h. Precipitation of DNA was performed by standing overnight at 4 °C in presence of 3 M Na-Acetate (pH 5.2) and isopropanol. The next day, the DNA was extracted by using DNA Extraction Reagent (SOLARBIO). The obtained DNA was washed with 500 μl 75% cold ethanol, dissolved in 40 μl 1 × TE buffer containing 0.1 mg/ml RNase A, and incubated for 30 min at 37 °C. Successful proximity ligation was confirmed by running 5 μl of the DNA on a gel. Finally, the resulting DNA was used for library construction and paired-end sequenced on the BGI DNBSEQ platform. Two biological replicates were used for each condition.

### 3C-seq data analysis

3C-seq reads were filtered and quality-evaluated using the software Cutadapt (61) and FastQC with the default parameters to generate clean reads. The clean reads were aligned independently to the reference genome (*D. radiodurans*: GCA_000008565.1) by Bowtie2 (62) using a very sensitive and iterative approach. Each read was then assigned to a restriction fragment, with uninformative events, such as self-circularized and uncut fragments discarded by filtering. Because these restrictive fragments presented unequal lengths, the genome was binned into 5 kb segments to generate the interaction matrix files. After correlation analysis, the two biological replicates were merged for subsequent analysis. The original contact matrices were then normalized using the sequential component normalization procedure (SCN) (63) to reduce the biases inherent resulting from the 3C experimental protocol.

CIDs were identified by the directionality index (DI), which is a measure that quantifies the degree of upstream or downstream contact bias for a genomic region (6). CID boundaries were defined as the area where the direction preference of bins shifted from negative values of upstream segments to positive values of downstream segments. Sequence motifs of CID boundaries were investigated in the PRODORIC database using MEME Suite (https://meme-suite.org). Significant intra-chromosome interactions in which both the *p*-value and *q*-value (adjusted *p*-value for Benjamini-Hochberg correction) were less than 0.05, and contact count > 2 were selected by FitHiC2 software (64).

### Reconstruction of the 3D chromosome model of *D. radiodurans*

The 3D chromosome models of *D. radiodurans* were reconstructed based on the SCN normalized interaction frequency matrix using the EVRC software (33). The EVRC program generates PDB files containing the spatial coordinates of each node in the 3D structure corresponding to a bin of the IF matrix. The coordinate file was visualized using PyMOL (The PyMOL Molecular Graphics System, Version 2.4.0 Schrödinger, LLC). It should be noted that, like all existing methods, EVRC cannot distinguish whether the interactions are intra- or inter-molecule contacts within each replicon since *D. radiodurans* is polyploid. Therefore, each model we construct corresponds to the average structure of multi-copied chromosomes. These structures, although not perfect, intuitively reflect some difficult-to-observe features of the contact map.

### Constructing genome-scale metabolic model for flux balance analysis

A draft metabolic model was constructed using the KBase Narrative Interface (www.kbase.us) and then manually filled the gaps. The final *D. radiodurans* model is termed *i*DR741 and includes 741 genes, 1148 reactions, and 1106 metabolites. The SBML file of the model was validated by the online SBML validator tool (http://sbml.org/Facilities/Validator/), and provided on GitHub. The transcriptomic data were integrated into the metabolic network by the E-Flux2 method (65) to construct models of *D. radiodurans* wild-type and Δ*ebfC* mutant. Metabolic modeling and flux balance analysis were performed using Cobra Toolbox v3.0 (66) in Matlab R2022b (Mathworks), and the solver was Gurobi 11.0.2 (www.gurobi.com).

## Data availability

3C-seq and RNA-seq FASTQ files are deposited in the Gene Expression Omnibus (accession no. GSE241498 and GSE241624). The scripts used for analyzing 3C data have been deposited to Github (https://github.com/mbglab/DR_3C_analysis).

## Supporting information

This article contains supporting information.

## Conflict of interest

The authors declare that they have no conflicts of interest with the contents of this article.
