# Supplementary material for: Spatial chromosome organization and adaptation of the radiation-resistant extremophile Deinococcus radiodurans
Source: J Biol Chem. 2024 Dec 10;301(1):108068. doi: 10.1016/j.jbc.2024.108068 (PMC11758949; doi:10.1016/j.jbc.2024.108068)
Supplement: Supporting information [file mmc1.pdf]

Spatial chromosome organization and adaptation of the radiation-resistant extremophile *Deinococcus radiodurans*

Qin-Tian Qiu, Cai-Yun Zhang, Zhi-Peng Gao, Bin-Guang Ma*

Hubei Key Laboratory of Agricultural Bioinformatics, College of Informatics,
Huazhong Agricultural University, Wuhan 430070, China

* Corresponding author. Tel & Fax: +86 2787280877.
E-mail address: mbg@mail.hzau.edu.cn (Bin-Guang Ma)

List of supporting information:

Figure S1
Figure S2
Figure S3
Figure S4
Figure S5
Figure S6
Figure S7
Figure S8
Figure S9
Figure S10
Figure S11
Figure S12
Figure S13
Figure S14
Table S1
Table S2
Table S3
Table S4

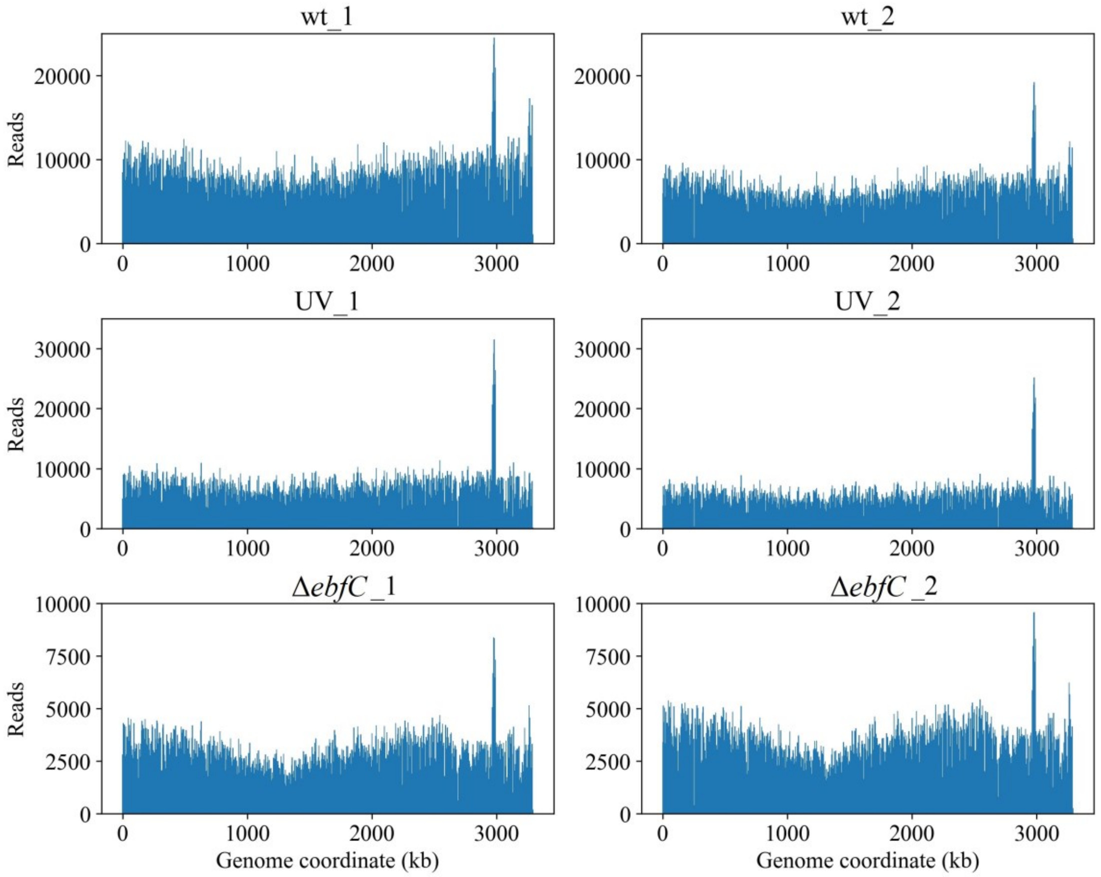

Figure S1. The coverage distribution of sequencing reads along the genome position for *D. radiodurans* 3C-seq samples. wt_1/ wt_2, UV_1/ UV_2 and Δ*ebfC*_1/ Δ*ebfC*_2 represent two biological replicates for wild-type, ultraviolet irradiation condition and Δ*ebfC* mutant strain, respectively.

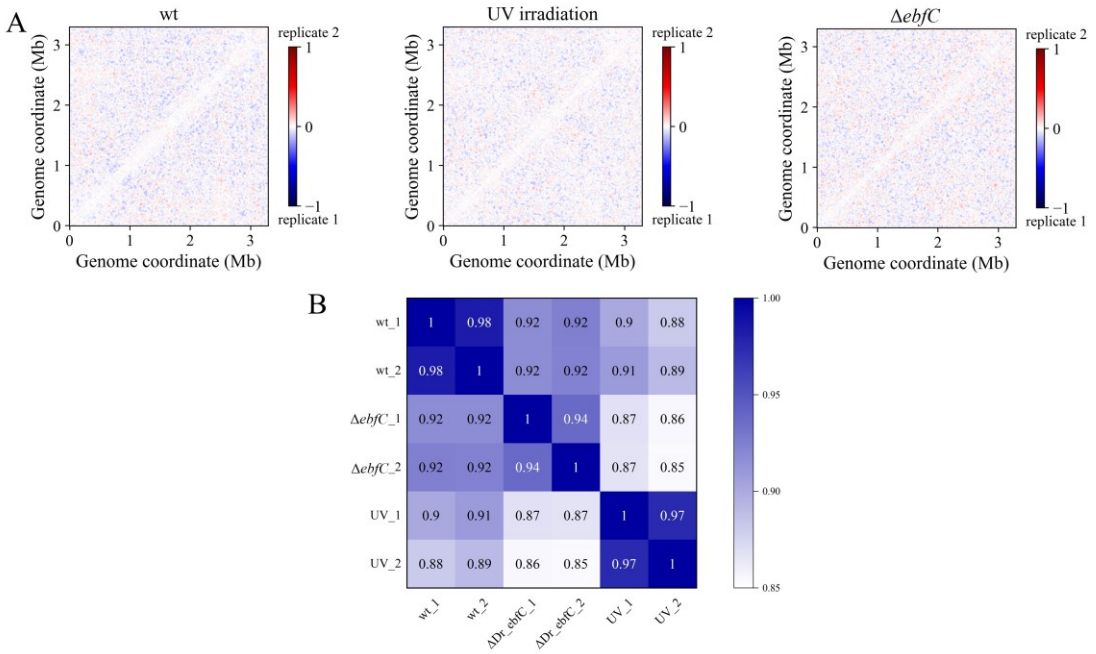

Figure S2. Analysis of experimental replicates. (A) Ratio of normalized contact maps of two independent replicates for wild-type, ultraviolet irradiation condition and Δ*ebfC* mutant strain, respectively. (B) The correlation heatmap.

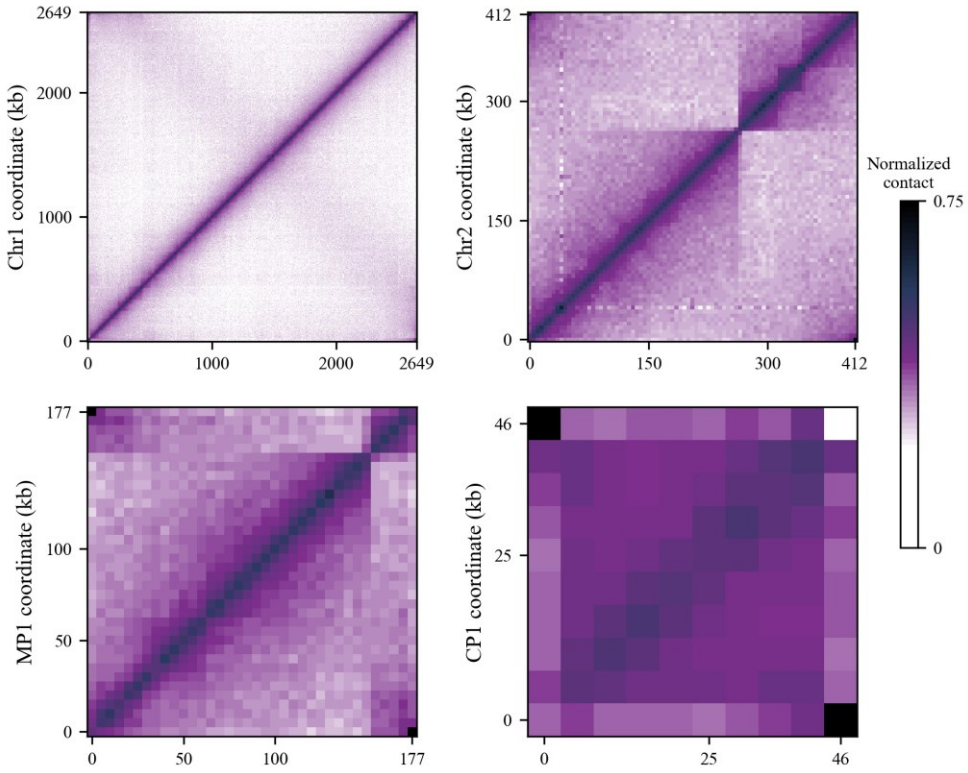

Figure S3. Chromosome contact map of the four replicons of *D. radiodurans.*

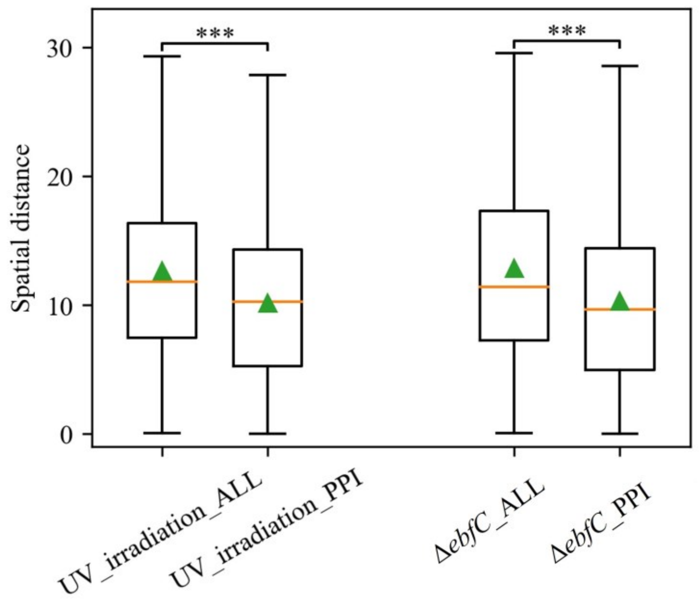

Figure S4. Boxplot of the spatial distance distribution between bin pairs in the 3D mode. The two groups (ALL vs. PPI) on the *x*-axis correspond to the distribution of spatial distance between all bins (ALL) and between the bins containing protein-protein interaction (PPI), respectively. The orange line indicates the median of the box; the green triangle indicates the average value of the box. ***, p-value < 0.001. UV_irradiation: ultraviolet irradiation condition; Δ*ebfC*: Δ*ebfC* mutant strain.

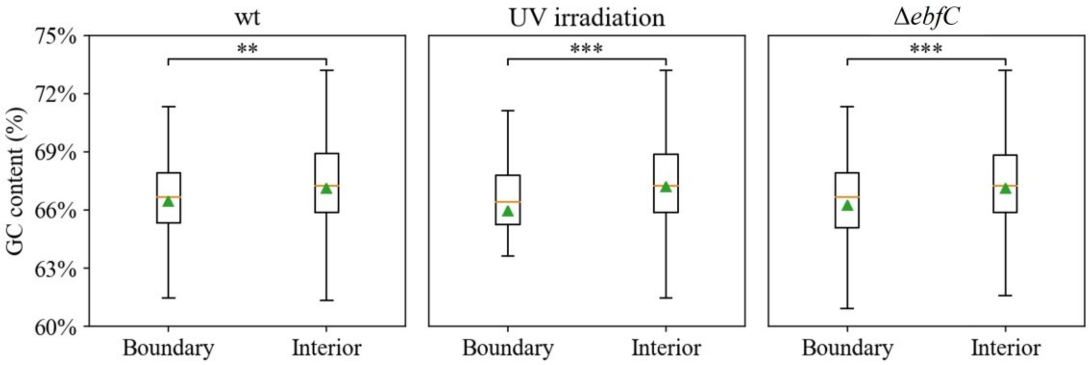

Figure S5. Box plots of GC content distributions corresponding different samples. The GC content of CID boundary is significantly lower than that of the CID interior. The orange line indicates the median of the box; the green triangle indicates the average value of the box. **, p-value < 0.01; ***, p-value < 0.001. wt: wild-type; UV irradiation: ultraviolet irradiation condition; Δ*ebfC*: Δ*ebfC* mutant strain.

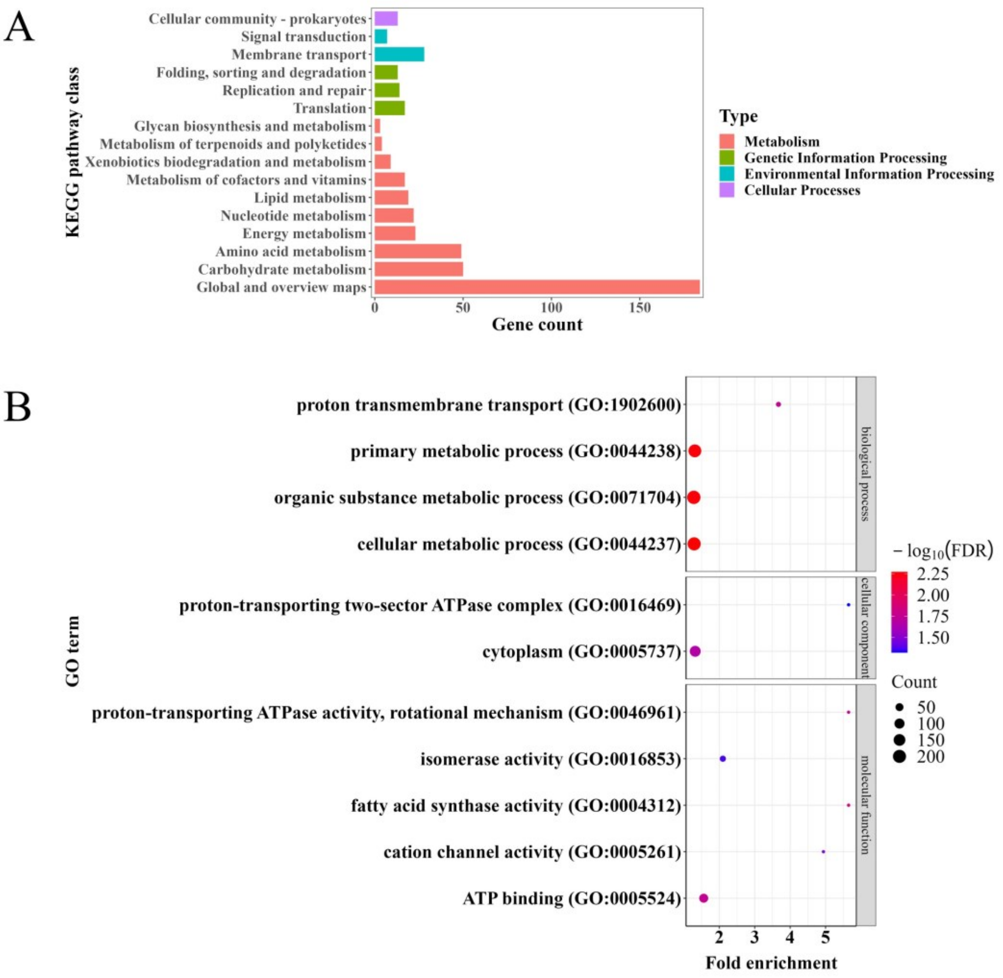

Figure S6. KEGG and GO enrichment analysis of genes localized in wild-type CID boundaries. (A) Kyoto Encyclopedia of Genes and Genomes (KEGG) pathway analysis of genes localized in CID boundaries. (B) Gene Ontology (GO) enrichment results of the CID boundary genes.

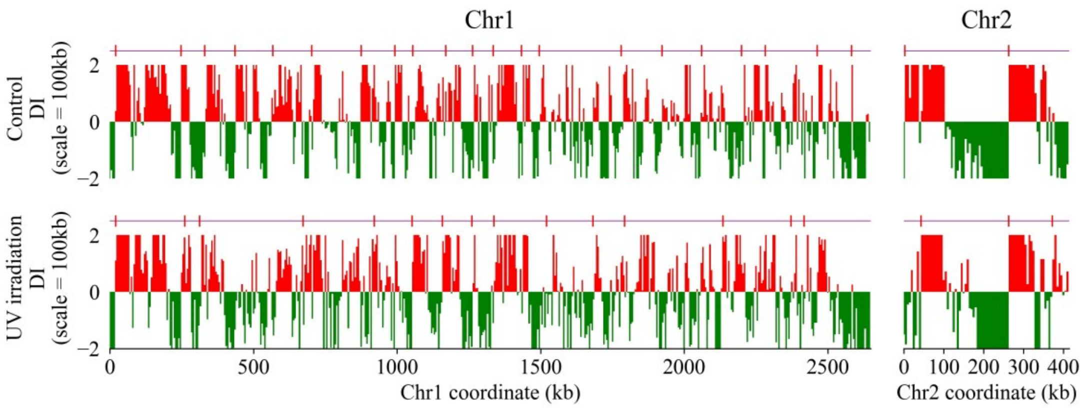

Figure S7. Domain boundaries characterized for the control and UV irradiation conditions using a DI analysis performed at a scale of 100 kb. Downstream (red) and upstream (green) biases are indicated. Significant boundaries defining CIDs are annotated with red vertical lines above the panel.

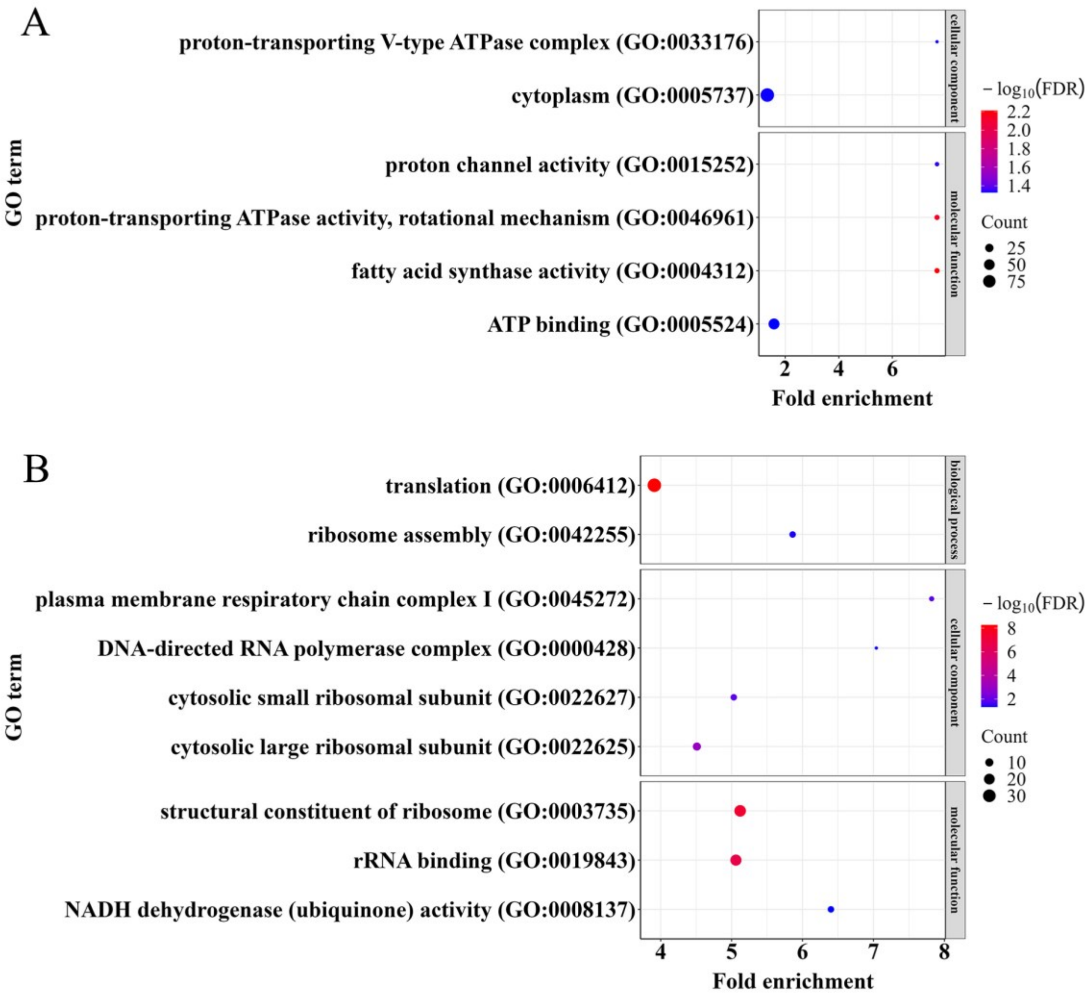

Figure S8. Comparison of GO enrichment terms of specific CID boundary genes under two conditions. (A), (B) GO enrichment results of the CID boundary genes for the control group and UV irradiation condition, respectively.

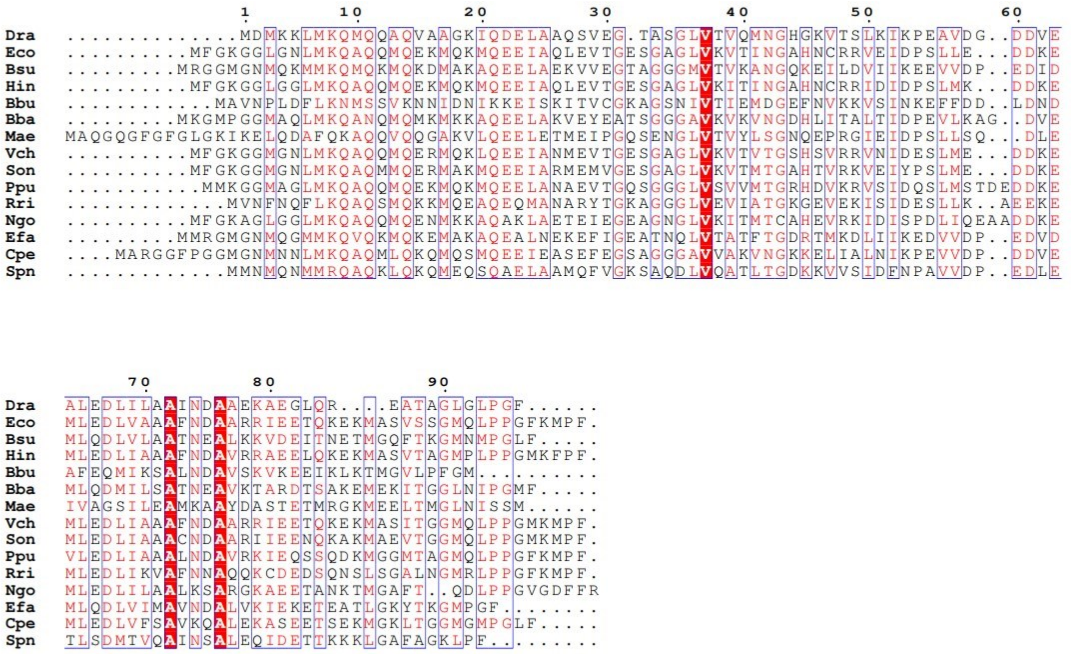

Figure S9. Multiple sequence alignment of YbaB/EbfC proteins from different bacterial species using ClustalW software. Identical amino acids are boxed as white characters on a red background, and similar amino acids as red characters on a white background. The selected species are *Deinococcus radiodurans* (Dra), *Escherichia coli* (Eco), *Bacillus subtilis* (Bsu), *Haemophilus influenzae* (Hin), *Borreliella burgdorferi* (Bbu), *Bdellovibrio bacteriovorus* (Bba), *Microcystis aeruginosa* (Mae), *Vibrio cholerae* (Vch), *Shewanella oneidensis* (Son), *Pseudomonas putida* (Ppu), *Rickettsia rickettsiae* (Rri), *Neisseria gonorrhoeae* (Ngo), *Enterococcus faecalis* (Efa), *Clostridium perfringens* (Cpe) and *Streptococcus pneumoniae* (Spn), respectively.

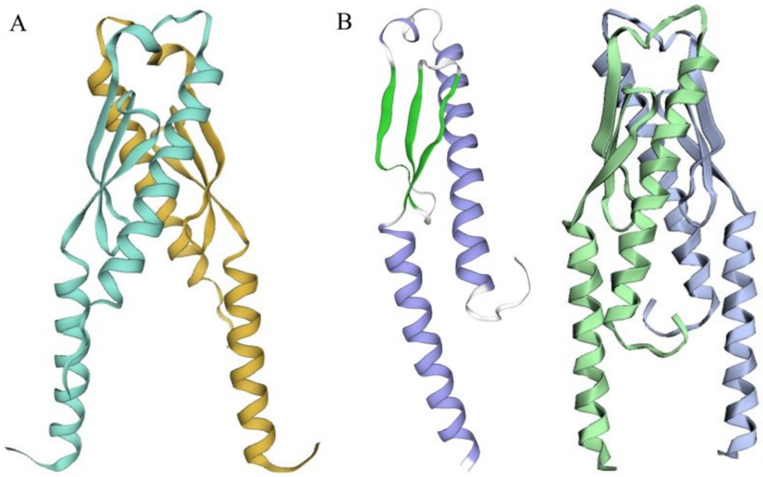

Figure S10. The tertiary structure of DrEbfC protein. (A) The predicted homodimer structure of DrEbfC protein using SWISS-MODEL. (B) The predicted monomer and homodimer structures of DrEbfC protein using AlphaFold2.

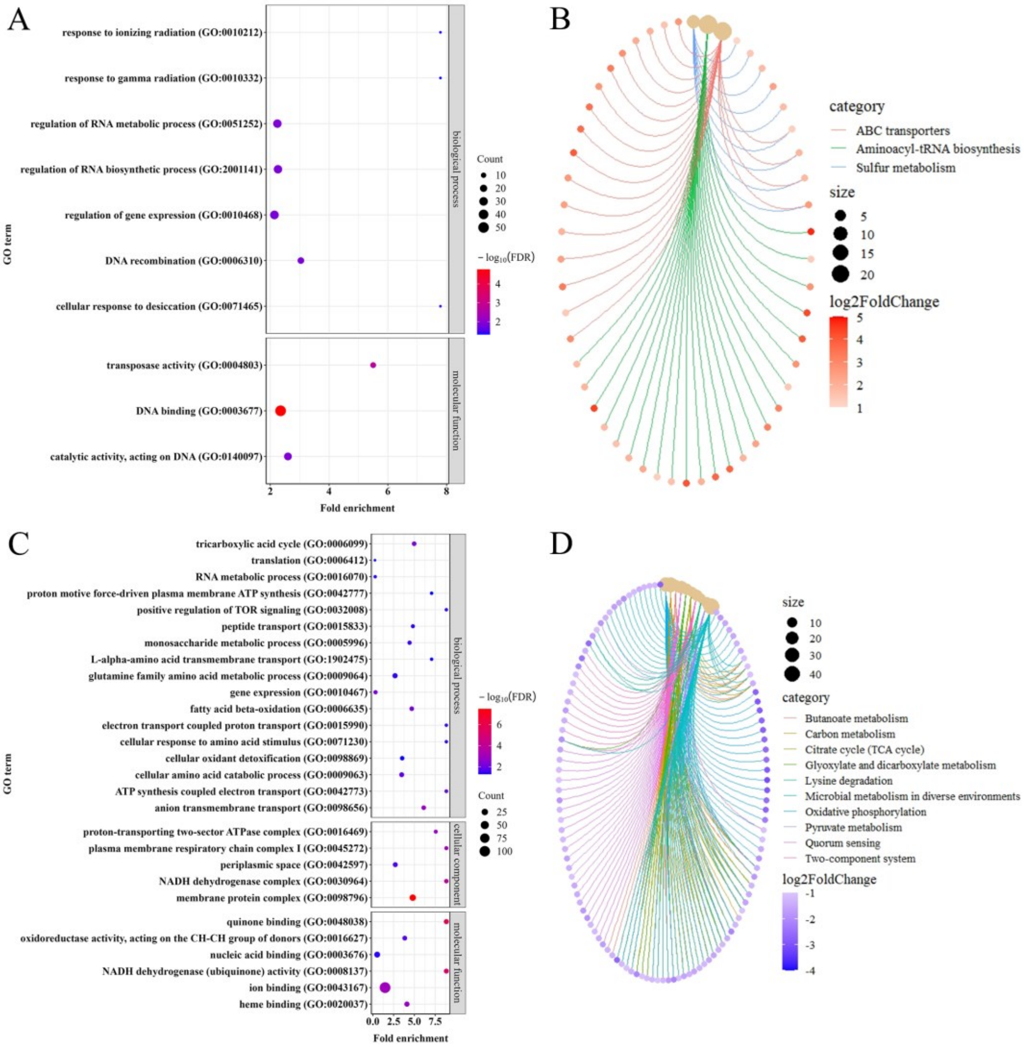

Figure S11. Enrichment results of differentially expressed genes (DEGs) in the transcriptional profiles of wild-type and Δ*ebfC* mutant strains. (A), (B) GO and KEGG enrichment results of the upregulated DEGs, respectively. (C), (D) GO and KEGG enrichment results of the downregulated DEGs, respectively.

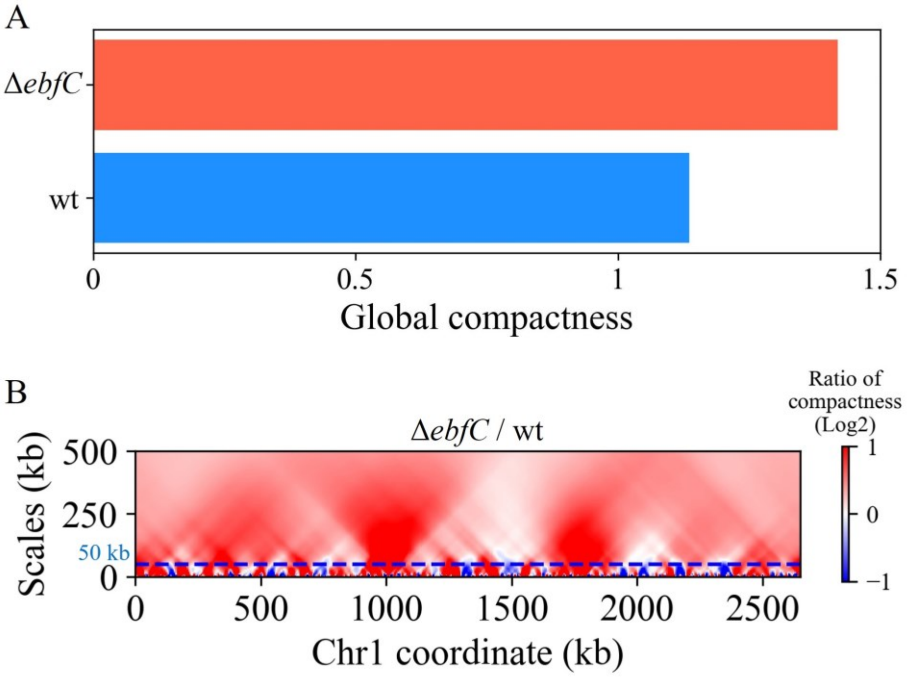

Figure S12. Calculation of global and local compactness of chr1 structure model. (A) Global compactness of Chr1 for two strains. (B) Ratio (Δ*ebfC* / wt) plot of the local compactness for each bin along Chr1. The *x*-axis indicates the position of the bin along the genome. The *y*-axis indicates the distance from the bin. A decrease or increase in the local compactness at the Δ*ebfC* compared with the wt is represented with a blue or red color, respectively. White color indicates no difference between the two strains.

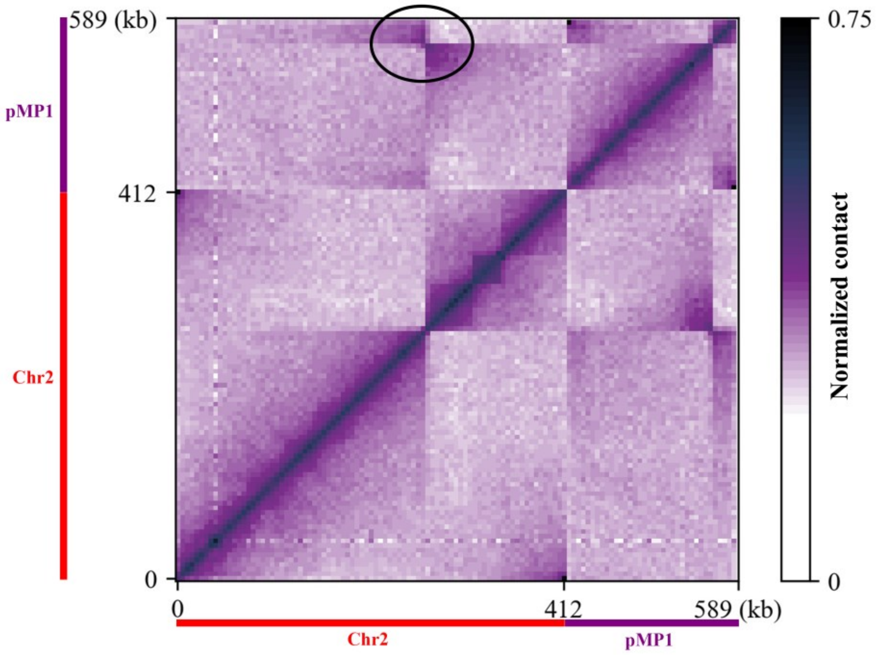

Figure S13. Contact map of the Chr2 and pMP1 of *D. radiodurans*. Chr2 and pMP1 are indicated by red and purple bars, respectively. A clear contact signal (recombination event) between Chr2 and pMP1 is indicated by a black circle.

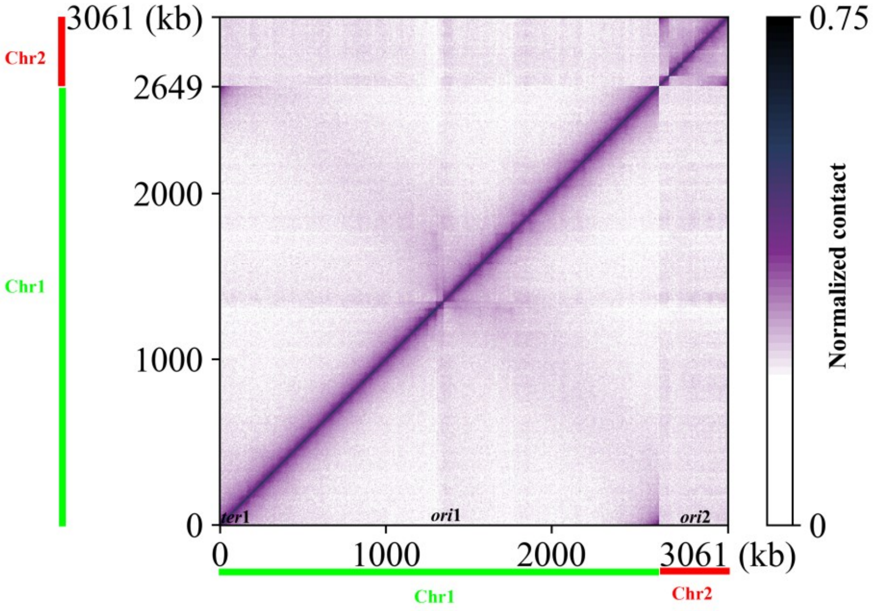

Figure S14. Contact map of the two chromosomes of *D. radiodurans*. Chr1 and Chr2 are indicated by green and red bars, respectively. To better visualize contacts in the origin region, the genome coordinates of two chromosomes are rearranged with the origin at the center and the two arms on either side. The positions of the origins (*ori*1 and *ori*2) and terminus (*ter*1) are indicated on the x axis.

Table S1. List of strains, plasmid, and primers used in this experiment

Name	Relevant characteristics, or sequence	Source
Strains		
D. radiodurans R1	Wild-type strain ATCC13939	Laboratory stock
Δ*ebfC*	R1 but *dr_0199::kan*	This study
Plasmid		
pRADK	*D. radiodurans* shuttle vector	Laboratory stock
Primers		
Δ*dr_0199*-p1	CACTTCCAGTTCGGCCACGTCC	This study
Δ*dr_0199*-p2	ACAGACGGATCCGACCGTCAGTCTAGCGGCT	This study
Δ*dr_0199*-p3	CATAGAAGCTTGTGTGACCGCGCTGGC	This study
Δ*dr_0199*-p4	CTTGCTCACGGTCTGGCGG	This study
kan-p5	CATAGAAGCTTCGTATTGTCGCCCTACATAT	This study
kan-p6	ACAGACGGATCCTAGAAAAACTCATCG	This study
Δ*dr_0199_test*-F	GCTTCGGGCTTGATTTTCAGG	This study
Δ*dr_0199_test*-R	CGGCTGGCAAGATTCAGGAC	This study

Table S2. Predicted motifs of transcription factors and NAPs in CID boundaries

Identifier	Width	E-value	Matched motif
motif_1	29	6.00E-15	MX000107 (OxyR), MX000207 (McbR), MX000095 (MhpR), MX000147 (MetJ), MX000177 (TreR)
motif_2	29	1.00E-12	MX000152 (GlnG), MX000103 (ExsA), MX000102 (Dnr), MX000095 (MhpR), MX000002 (Anr), MX000186 (AlgZ), MX000178 (TorR), MX000111 (RpoN), MX000070 (SigE), MX000163 (Lrp), MX000171 (RhaS), MX000132 (FliA), MX000190 (FNRL), MX000192 (NnrR)
motif_3	15	4.50E-09	MX000192 (NnrR), MX000095 (MhpR), MX000152 (GlnG), MX000177 (TreR), MX000007 (ArgR), MX000180 (HydG), MX000119 (CpxR), MX000111 (RpoN), MX000002 (Anr), MX000101 (AlgR), MX000021 (Spo0A), MX000190 (FNRL), MX000046 (ComA)
motif_4	21	1.90E-07	MX000095 (MhpR), MX000152 (GlnG), MX000192 (NnrR), MX000042 (RegR), MX000033 (AlgU), MX000163 (Lrp), MX000030 (DegU), MX000164 (Lrp), MX000208 (PrrA), MX000186 (AlgZ), MX000180 (HydG), MX000034 (AlgU), MX000111 (RpoN), MX000107 (OxyR), MX000126 (FlhD2C2), MX000069 (SigE)
motif_5	20	1.90E-07	MX000180 (HydG), MX000042 (RegR), MX000101 (AlgR), MX000152 (GlnG), MX000192 (NnrR), MX000095 (MhpR), MX000033 (AlgU), MX000111 (RpoN), MX000034 (AlgU), MX000124 (FadR), MX000046 (ComA), MX000115 (AraC), MX000208 (PrrA), MX000186 (AlgZ), MX000178 (TorR), MX000002 (Anr), MX000126 (FlhD2C2)
motif_6	20	2.00E-07	MX000095 (MhpR), MX000177 (TreR), MX000001 (MexR), MX000002 (Anr), MX000183 (TrpR), MX000008 (GlpR), MX000171 (RhaS), MX000033 (AlgU), MX000208 (PrrA), MX000101 (AlgR), MX000126 (FlhD2C2), MX000070 (SigE), MX000172 (RhaR)
motif_7	26	1.30E-06	MX000171 (RhaS), MX000101 (AlgR), MX000065 (SigD), MX000095 (MhpR), MX000137 (LacI), MX000148 (ModE), MX000115 (AraC), MX000203 (DevR), MX000034 (AlgU), MX000001 (MexR), MX000177 (TreR), MX000104 (FleQ), MX000208 (PrrA), MX000139 (MalT)
motif_8	29	5.40E-06	MX000203 (DevR), MX000186 (AlgZ), MX000107 (OxyR), MX000006 (SpoIIID), MX000103 (ExsA), MX000195 (YsiA), MX000130 (GalS), MX000127 (FhlA), MX000193 (KdgR), MX000191 (PpsR)
motif_9	21	1.70E-05	MX000025 (DinR/LexA), MX000021 (Spo0A), MX000151 (NhaR), MX000095 (MhpR), MX000070 (SigE), MX000097 (Mlc), MX000107 (OxyR), MX000183 (TrpR), MX000207 (McbR), MX000172 (RhaR), MX000147 (MetJ), MX000176 (YdiH)

Table S3. List of genes belonging to the PprI/DdrO regulon in *D. radiodurans*.

Gene ID	Log_2FC	*P*-adj	Direction	Locus tag	Function description
		Replication, recombination, and repair (17)			
DR_RS00525	0.76	3.29E-02	ns	DR_0100	single-stranded DNA-binding protein SSB
DR_RS02185	3.85	9.58E-26	up	DR_0423	single-stranded DNA-binding protein DdrA
DR_RS00370	3.05	1.95E-23	up	DR_0070	single-stranded DNA-binding protein DdrB
DR_RS00015	3.10	4.24E-22	up	DR_0003	DNA damage response protein DdrC
DR_RS01690	2.92	1.49E-10	up	DR_0326	DNA damage response protein DdrD
DR_RS09060	-0.80	1.02E-02	ns	DR_1771	excinuclease ABC subunit UvrA
DR_RS11695	-0.35	1.05E-01	ns	DR_2275	excinuclease ABC subunit UvrB
DR_RS12030	0.55	1.00E-01	ns	DR_2340	recombinase RecA
DR_RS09795	-0.41	5.79E-02	ns	DR_1913	DNA gyrase subunit A
DR_RS04680	-0.96	5.67E-04	ns	DR_0906	type IIA DNA topoisomerase subunit B
DR_RS03100	0.65	2.31E-02	ns	DR_0596	Holliday junction branch migration DNA helicase RuvB
DR_RS05355	2.09	6.85E-10	up	DR_1039	DNA mismatch repair protein MutS
DR_RS09810	0.25	4.26E-01	ns	DR_1916	ATP-dependent DNA helicase RecG
DR_RS09740	2.12	1.68E-09	up	DR_1902	ATP-dependent RecD-like DNA helicase
DR_RS10620	-0.20	4.14E-01	ns	DR_2069	NAD-dependent DNA ligase LigA
DR_RS12020	1.80	5.56E-07	up	DR_2338	CinA family mononucleotide deamidase-related protein
DR_RS15335	1.30	5.05E-07	up	DR_A0346	DNA repair protein PprA
		Translation and post-translational modification (4)			
DR_RS00725	0.17	6.45E-01	ns	DR_0139	GTPase HflX
DR_RS11140	0.24	3.20E-01	ns	DR_2174	leucine--tRNA ligase
DR_RS11600	2.45	1.52E-05	up	DR_2255	GNAT family N-acetyltransferase
DR_RS12555	0.04	8.97E-01	ns	DR_2441	GNAT family N-acetyltransferase
		Metabolism and metabolic transport (5)			
DR_RS01110	-0.24	4.21E-01	ns	DR_0217	sulfurtransferase
DR_RS02900	-0.50	1.74E-01	ns	DR_0561	extracellular solute-binding protein
DR_RS06680	1.51	2.01E-06	up	DR_1297	DUF808 domain-containing protein
DR_RS11605	0.27	3.53E-01	ns	DR_2256	transketolase
DR_RS14970	-1.68	5.23E-09	down	DR_A0275	cytochrome c
		Unknown function (7)			
DR_RS01120	-0.60	5.14E-02	ns	DR_0219	hypothetical protein
DR_RS03565	0.58	1.97E-02	ns	DR_0685	DUF11 domain-containing protein
DR_RS05905	2.17	2.65E-08	up	DR_1143	hypothetical protein
DR_RS08030	-1.23	3.02E-04	down	DR_1571	ABC transporter substrate-binding protein
DR_RS11135	1.60	6.34E-06	up	DR_2173	DUF1963 domain-containing protein
DR_RS14425	0.46	1.31E-01	ns	DR_A0165	hypothetical protein
DR_RS16300	1.06	9.40E-03	up	DR_C0023	hypothetical protein
		Ambiguous (3)			
DR_RS16270	3.62	1.09E-19	up	DR_C0017	Tn3 family transposase
DR_RS06675	0.68	0.004257	ns	DR_1296	IS5-like element ISDra5 family transposase
DR_RS16355	0.50	0.037748	ns	DR_C0033	IS5-like element ISDra5 family transposase

Note: The three columns of Log_2FC, *P*-adj, and Direction are derived from the

differential expression analysis of mutant and wild-type cells. Up and down indicate |fold change| > 2 and *P*-adj < 0.05, respectively. Ns indicates |fold change| < 2 or *P*-adj > 0.05.

Table S4. Genes involved in oxidative resistance

Gene ID	Log_2FC	*P*-adj	Gene name	Locus tag	Function description
DR_RS10235	-2.08	1.27E-08	*katA*	DR_1998	catalase
DR_RS14880	-1.28	1.46E-04	*katE*	DR_A0259	catalase
DR_RS14330	-1.44	7.11E-06		DR_A0146	catalase family protein
DR_RS06595	-1.53	7.78E-07	*sodA*	DR_1279	superoxide dismutase [Mn]
DR_RS07910	-1.46	2.39E-07		DR_1546	superoxide dismutase family protein
DR_RS07020	-1.80	3.94E-06	*pdxT*	DR_1366	pyridoxal 5'-phosphate synthase glutaminase subunit PdxT
DR_RS07025	-1.86	1.63E-06	*pdxS*	DR_1367	pyridoxal 5'-phosphate synthase lyase subunit PdxS
